# Organisation of event files–can temporal factors influence stimulus–response binding and retrieval?

**DOI:** 10.1007/s00426-026-02394-3

**Published:** 2026-11-11

**Authors:** Anna Scotts, Malte Möller, Ruyi Qiu, Iring Koch, Susanne Mayr

**Affiliations:** 1 Psychology and Human-Machine Interaction, University of Passau, Passau, 94032 Germany; 2 Department of Psychology, Hunan University of Chinese Medicine, Changsha, Hunan Province 410208 China; 3 Psychology I: Cognitive and Experimental Psychology, RWTH Aachen University, Aachen, 52062 Germany

## Abstract

This paper explores how temporal factors, that is, veridical time intervals and symbolic (adverbial) references to time derived from event segmentation theory, influence the retrieval of common stimulus and response representations, so called *event files*. In a series of four experiments, we examined temporal factors in their role for event file retrieval by implementing time intervals (Experiment 1, *N* = 64), temporal adverbials (Experiment 3, *N* = 70), and a combination of both (Experiment 4, *N* = 74). Specifically, we introduced a temporal manipulation between event file binding and retrieval—that is, between the sequential presentations—to investigate whether the likelihood of event file retrieval is reduced after long intervals or adverbials referring to a long period. Experiment 1 showed that long time intervals reduced the after-effects of S-R binding. In Experiment 2 (*N* = 71), we confirmed that the temporal adverbials used in Experiments 3 and 4 are generally capable of inducing subjective event boundaries when segmentation is task-relevant as in a typical event segmentation task. However, we did not find any effects suggesting that the mere semantic reference to time intervals in Experiment 3 has the same effect as veridical (i.e. physical) time intervals. This also held for semantic reference to time intervals that have been associated with actual time intervals (Experiment 4). Thus, the findings are more consistent with an interpretation of decay, in which the passage of veridical time gradually weakens the strength of the memory trace representing an event file, than with event segmentation, in which symbolic temporal information should induce an event boundary and thereby reduce the likelihood of retrieval.

**Supplementary information:**

The online version contains supplementary material available at https://doi.org/10.1007/s00426-026-02394-3.

## Introduction

Human beings presumably create links between stimuli and their responses to these stimuli resulting in a common episodic representation referred to as an *event file, stimulus*–*response binding* or a *stimulus-response (S-R) episode* (for overviews, see Theory of Event Coding; TEC; Hommel, 2004 and the Binding and Retrieval in Action Control framework; BRAC; see Frings et al., 2020). Re-encountering any feature of an event file presumably retrieves the whole episode, leading to impaired performance if the retrieved information mismatches a subsequent stimulus or response. In other words, when either stimulus information or response requirements change between successive presentations, processing is impaired, leading to increased response times and/or error rates as compared with situations in which the stimulus and the response both repeat or both change (so-called *partial repetition costs, PRCs*). S-R binding and retrieval is mainly investigated in paradigms with sequential presentations, with S-R binding taking place in a so-called *prime* followed by the retrieval of event files in a so-called *probe* (Frings et al., 2020). Previous studies suggest that binding and retrieval of event files can be modulated by several factors (for a detailed outline, see Frings et al., 2020), some of which are closely related to factors that are described in event segmentation theory (EST; Zacks, 2020; Zacks et al., 2007).

### Event segmentation theory

Following the EST, a so-called *event* is considered a segment of time at a given location that is perceived to have a beginning and an end (Zacks & Tversky, 2001). Humans are thought to organise the continuous stream of ongoing activity into separable events by either unitising or segmenting them into parts (Franklin et al., 2019; Richmond & Zacks, 2017; Zacks, 2020; Zacks et al., 2007). For example, depending on the current focus of interest, the event of “making tea” can be split up into separate smaller events like opening a cupboard, taking out a cup, adding a tea bag, filling the kettle with water. The EST suggests that this perception of events is driven by prediction. The human cognitive system is equipped to detect and track regularities (Buchner & Steffens, 2001; Richmond & Zacks, 2017) in the environment and thus use previous and current information to attempt to predict the near future (Zacks, 2020). Further, EST assumes that the predicted outcomes are stored as a “working event model” and the content of that model is continuously compared to the actual environment. Importantly, an event boundary is experienced when the predictions from the model become sufficiently inaccurate, ultimately resetting the model. In other words, the current event model is flushed or updated at event boundaries (Radvansky & Zacks, 2017; Zacks et al., 2007). Experiencing an event boundary has consequences for human perception and behaviour as several studies suggest. For example, DuBrow and Davachi (2016) found that associations among information presented *within* an event are stronger than among information presented *across* an event boundary. Similarly, Speer and Zacks (2005) demonstrated that information within an event is more easily retrieved than information separated by an event boundary. Furthermore, memory for recently mentioned objects is poorer after reading sentences that indicate a shift in time or space (Rinck & Bower, 2000). Several factors (so-called *organisation factors* like temporal or spatial information, as well as subjective goals) influence the occurrence of event boundaries (for a recent review, see Zacks, 2020).

### Organisation factors and event files

More recent iterations of the EST framework propose a close connection between event perception, language, memory, and action control, introducing the broader “event cognition” framework (Radvansky & Zacks, 2014, 2017; Zacks, 2020). Following this notion, there might be an intersection between the two concepts “event file” from the BRAC framework and “event” from the EST. In fact, both theoretical perspectives assume that stimulus and response information is stored in a common representation (Hommel, 2004; Radvansky & Zacks, 2017). Given these similarities, it might be assumed that the integration and retrieval of an event file (as postulated by the TEC/BRAC framework) can be affected by event boundaries as described in EST.

In previous studies, organisational factors from EST, such as contextual change (Mayr et al., 2018; Moeller et al., 2025; Qiu et al., 2023) or goals (Fournier & Gallimore, 2013), were found to modulate S-R binding and/or retrieval processes. In two auditory selection experiments using sequential prime-probe paradigms, Qiu et al. (2023) demonstrated an effect of event boundaries on event file binding: In addition to target and distractor sounds, sine tones were presented as context information during the prime, whereas no explicit context sound was presented during the probe. In the so-called *common context* condition, the context started with the prime sounds and ended with the prime response. In the *changing context* condition, the context started with the prime sounds but changed to another tone frequency after the offset of the prime sounds, implying that prime stimuli and prime response were separated by a context change, presumably constituting an event boundary. Results from both experiments revealed a smaller S-R binding effect in the changing context condition than in the common context condition, suggesting that event boundaries created by contextual change weaken the integration of event files, and, as a consequence, lessen its after-effects.

Another way to induce event boundaries consists of using temporal organisation factors, as has been shown in studies investigating event cognition (DuBrow & Davachi, 2016; Speer & Zacks, 2005). Put simply, temporal manipulations representing long periods of time between prime and probe may induce event boundaries that reduce the likelihood of retrieval of event files.

### Effects of temporal manipulations on event files

Throughout this paper, temporal factors refer to two distinct ways of manipulating time: veridical time, that is, actual elapsed intervals, and symbolic reference to time, such as temporal adverbials. Effects of temporal manipulations have been reported in studies on event segmentation. The effect of time on the occurrence of event boundaries might be explained by the gradual change of context during a period of time: Context can change rather abruptly (i.e., *shift*) or change slowly but continuously over time (i.e. *drift*, DuBrow et al., 2017). According to this, it might be assumed that context drifts over the duration of a time interval, thereby increasing the prediction error until (given that the interval is sufficiently long) it becomes too large, and an event boundary is induced. For example, in a study by Paine and Gilden (2013), a prime-probe paradigm was used and, to manipulate an event boundary within this task, the authors varied the duration of intertrial intervals (which were either predictable or unpredictable) between the prime and the probe. Results showed that the partial repetition costs were decreased at long predictable intervals, that is, when the prime and the probe were placed across the (assumed) event boundary compared to short intervals, when prime and probe were (presumably) placed within the same event.

Effects of temporal manipulations have also been found in binding and retrieval research. It is generally assumed that these effects attribute the decline of PRCs over increasing intervals to the gradual weakening of memory traces over time, such that event files become less retrievable the longer the interval following their formation (Frings et al., 2026; Hommel & Colzato, 2004; Hommel & Frings, 2020). The reduction of PRCs over increasing intervals has been interpreted as evidence that event files disintegrate over time as a function of spontaneous decay (Hommel & Frings, 2020). Empirically, event files have been found to remain retrievable for at least four seconds when a time interval separates prime and probe (Hommel & Colzato, 2004), with after-effects of binding disappearing at around five seconds as intervals are systematically extended (Frings et al., 2026; Hommel & Frings, 2020). The presumed decay appears to be influenced by attention: bindings involving attended task-relevant stimuli have been found to be stronger and longer lasting than distractor bindings involving unattended task-irrelevant stimuli (Frings et al., 2022), and event files have been found to be more persistent across intervals when attentional resources were directed toward stimulus features during encoding (Hommel & Colzato, 2004).

Most importantly for the present purpose, there are studies showing that an event boundary can be created based on temporal manipulations, even when the actual time of the interval has been controlled for: previous studies on the EST have shown that an adverbial referring to a longer period of time induces an event boundary, whereas an adverbial referring to a shorter period does not (Ezzyat & Davachi, 2011; Speer & Zacks, 2005; Zacks et al., 2007), indicating an impact of linguistic information on formation of events (Baker & Levin, 2015; Faber et al., 2018). In a study by Ezzyat and Davachi (2011), participants read narratives that either contained the temporal adverbial “immediately after” or “after a while”, referring to “short” or “long” periods of time, respectively. Results of a cued-recall test showed impaired episodic memory for sentences separated by the adverbial “after a while” as compared with “immediately after”. The pattern was interpreted as evidence that mnemonic binding for sentences separated by a boundary (“after a while”) was weaker than for sentences not separated by a boundary (“immediately after”).

Ezzyat and Davachi (2011) also noted that much of the EST research uses narrative paradigms, in which mental models (or event models) are prominent (Van Dijk & Kintsch, 1983). In these studies, event boundaries are induced through spatial and temporal shifts in story-like settings. For instance, participants read narratives describing everyday events (e.g., taking photos) and indicated when one activity ended and another began (Speer & Zacks, 2005). If recently encountered information is upheld in event models until it is flushed at event boundaries, information in a narrative also becomes less accessible when presented after a temporal event boundary compared to when presented without a temporal event boundary (Speer & Zacks, 2005). This effect is not due to the veridical time elapsed since the information was encountered (Ezzyat & Davachi, 2011; Rinck & Bower, 2000; Speer & Zacks, 2005).

### Rationale of the present experiments

This paper investigates the role of temporal factors in event file retrieval. Two accounts of how temporal manipulations might modulate the likelihood of retrieval are considered. According to the event segmentation account, temporal factors affect retrieval by inducing event boundaries that partition experience into discrete episodes, thereby reducing the accessibility of representations formed prior to the boundary. According to a decay-based account, the passage of veridical time gradually weakens the memory trace representing a prior event file, regardless of whether an event boundary has been experienced. Importantly, only the event segmentation account makes predictions with respect to the role of symbolic temporal information: If the retrieval of event files can be modulated by an induced event boundary, temporal adverbials referring to a long period of time should modulate PRCs in a similar manner to long time intervals, given that such adverbials have been shown to induce event boundaries in narrative paradigms (Ezzyat & Davachi, 2011; Speer & Zacks, 2005). The decay-based account, by contrast, predicts that only veridical time should reduce PRCs, as symbolic references to time are not thought to alter the actual strength of the underlying memory trace.

In this series of experiments, we implemented temporal manipulations by means of veridical time (Experiment 1), and symbolic reference to time (Experiment 3), and a combination of both (Experiment 4) to investigate whether the likelihood of event file retrieval is affected. We incorporated elements from the narrative paradigm (Ezzyat & Davachi, 2011; Speer & Zacks, 2005) in the well-established S1R1-S2R2 task (Hommel, 1998) to improve the effectiveness of the temporal adverbial manipulation. Experiment 2 tested whether the symbolic reference to time in the form of temporal adverbials affects the subjective report of an event boundary with the sentences used in Experiments 3 and 4, thereby providing a manipulation check on the event boundary-inducing potential of the temporal adverbials independent of their effect on S-R binding. The pre-registered hypotheses for Experiments 1, 3 and 4 were derived from the event segmentation account, and accordingly we predicted that both long time intervals and temporal adverbials referring to a long period would reduce the likelihood of event file retrieval.

## Experiment 1

**Fig. 1:**
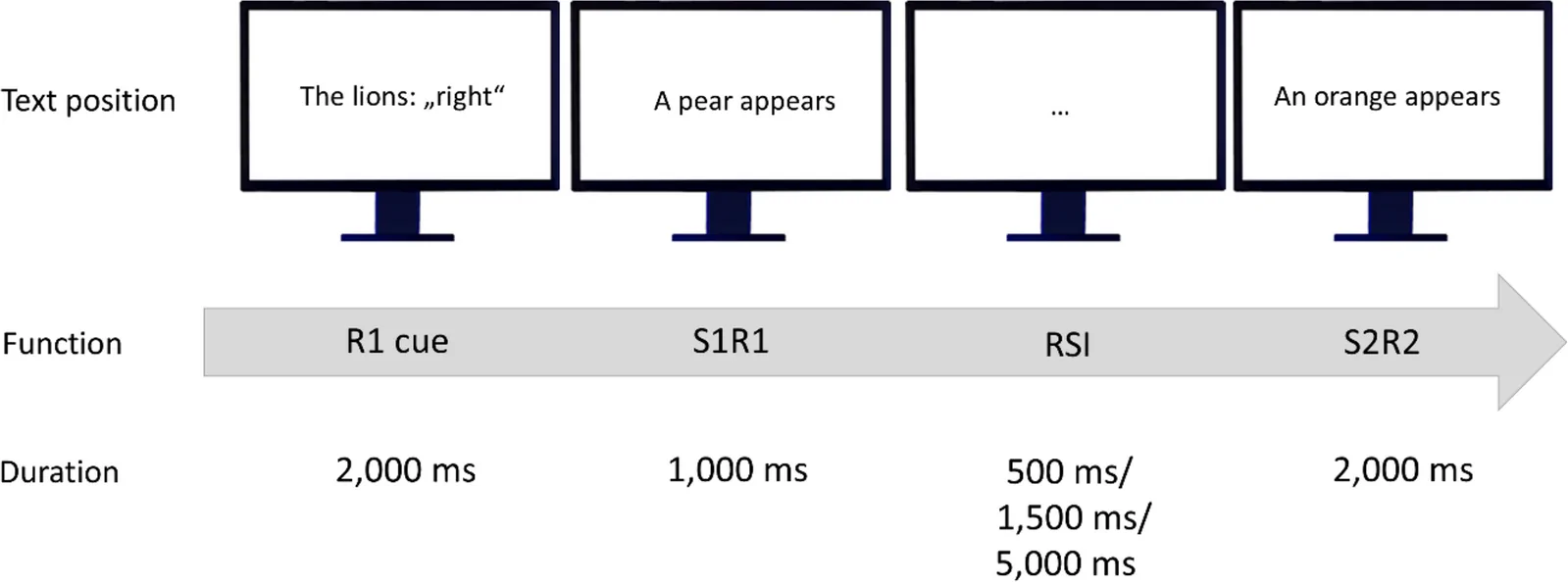
Trial sequence of Experiment 1. *Note.* On each trial, a response cue first indicated which key to press for R1 (left or right), but participants had to withhold that response until the first stimulus (S1) appeared, at which point they executed the cued response (R1) regardless of which stimulus it was. After the response-stimulus interval (RSI), the second stimulus (S2) appeared, and participants responded (R2) according to a fixed stimulus–response mapping rather than a cue. S1 (S2) refers to the first (second) stimulus, respectively. Responses to the stimuli (R1 for S1 and R2 for S2) are signalled by the cue (for R1) or are guided by the response rule (for R2). During RSI, three dots appeared successively with a time interval of 100 ms between each dot and disappeared afterwards

Experiment 1 had two goals: First, we incorporated elements from the narrative paradigm (Ezzyat & Davachi, 2011; Speer & Zacks, 2005) in the well-established S1R1-S2R2 task (Hommel, 1998) to replicate the common after-effects of event file binding in this paradigm. Specifically, narrative texts were embedded in the task to create an impression of a story-telling role-play game (see Fig. 1). This was necessary as Experiment 3 and 4 employed temporal adverbials to create an event boundary. Moreover, Experiment 1 tested whether increasing time intervals reduces the likelihood of event file retrieval. Event files are reflected by partial repetition costs which is indicated by longer response times and/or larger error rates in partial repetitions compared to full or no repetitions. On a statistical level, this is reflected by the interaction between stimulus and response relations between successive presentations. Therefore, we used response-stimulus intervals (RSIs) that varied across three levels (500 ms, 1,500 ms, 5,000 ms) between the prime and probe to manipulate binding and retrieval of event files.

### Method

#### Participants

A previous experiment by Hommel and Frings (2020) which systematically varied the onset interval between the S1 and the S2, observed a moderate to large modulation (*f* = 0.62, in terms of Cohen, 1988) of the S-R interaction (which is represented as partial repetition costs, PRCs) by the interval factor. Given the changes made to the design, such as using narrative texts instead of uppercase letters and RSIs instead of SOAs in Experiment 1 we expected to obtain a medium modulation effect of *f* = 0.25 only. Power calculations were performed using G*Power 3.1 (Faul et al., 2009, MANOVA: Repeated measures, within factors, one group, three measurements). For desired power of 95%, and an assumed correlation of ρ = .3 among the measures of the PRCs, data had to be collected from 61 participants.

Participants were recruited at the University of Passau and received either a 9 € Amazon voucher or course credit for their participation. A total of 64 (58 women, 6 men) participants were recruited whose age ranged from 18 to 39 years (*M* = 22.1, *SD* = 3.2). With the final sample size, the achieved power^1^ (.960) was slightly higher than originally planned for.

#### Transparency and openness

We comply with the TOP guidelines. All studies were pre-registered at aspredicted.org (for Experiment 1, see #150109 https://aspredicted.org/qdbq-fz3g.pdf). Data for the four experiments were collected in the years 2023, 2024 and 2025.

#### Materials and task

The general task for Experiments 1, 3 and 4 was modelled after a typical S1R1-S2R2 paradigm (Hommel, 1998, 2005). Each trial started with the presentation of a textual response cue (i.e., the written German words “*Die Löwen: links”* [“The lions: left”] or “*Die Löwen: rechts”* [“The lions: right”] indicating the to-be-executed response (R1, left or right button press). However, participants had to postpone their response until the first stimulus (S1, the written German words “*Eine Birne erscheint”* [“a pear appears”] or “*Eine Orange erscheint”* [“an orange appears”] appeared. Participants were asked to merely respond to the S1 as indicated by the response cue. The relationship between the sentence presented as S1 and the response required in R1 was completely arbitrary. The mapping of the response keys was spatially compatible (“f” for left, “j” for right) for all participants. Participants were instructed to use their index fingers of each hand to press the keys. The same sentences were used as second stimulus (S2), so that (depending on the condition) S1 and S2 could match or mismatch. The response (R2) to the S2 was not signalled in advance. Instead, the response to the S2 was governed by a rule (e.g., “pear” assigned to the left key and “orange” assigned to the right key or vice versa). The key mapping for the R2 in response to the S2 was counterbalanced across participants. The trial set was generated as follows: First, all possible combinations between R1 cue (“left” vs. “right”), S1 (“orange” vs. “pear”), and S2 (“orange” vs. “pear”) were created, resulting in a total of 8 trials. Among these trials, two unique trials belonged to each combination of stimulus and response relation: Stimuli and responses could either repeat or change between S1 and S2, and R1 and R2, respectively. In *Stimulus Repetition Response Repetition* trials, the stimulus repeated between S1 and S2 (e.g., “pear” and “pear”) and the response repeated between R1 and R2 (both times “j” key). In *Stimulus Repetition Response Change* trials, the stimulus repeated, but the response changed. In *Stimulus Change Response Repetition* trials, the stimulus changed, but the response repeated. Finally, in *Stimulus Change Response Change* trials, the stimulus and the response changed. Then, the set was combined with each of the three levels of the RSI factor. The resulting 24 trial types were presented eight times (in random order), creating the final set of 192 trials with 16 trials per cell. The RSIs varied across three levels (500 ms, 1,500 ms, or 5,000 ms). At the start of the RSI, three successively appearing dots were presented in 100 ms intervals.

### Experimental design

#### Procedure

All experiments were created using PsychoPy (Peirce et al., 2019) and were hosted online on the Pavlovia platform (https://pavlovia.org). The experimental code was transformed into PsychoJS code and required the use of the “Firefox” browser. After giving informed consent via a mouse click, participants were introduced to the task that was embedded in a fantasy-style story called the “Trial of the Lions”. Participants were instructed to take part in a magical tournament passing a gate that was secured by two stone lions. The participants were informed that the lions would test their abilities in terms of speed and judgment. Following the basic logic of a typical S1R1-S2R2 task, the lions would give them directional cues to test their speed and show them the two favourite fruits of the city’s royal couple to test their judgement abilities. To familiarize with the task, participants underwent two practice blocks. The first practice block (6 trials) was to familiarize participants with the sequence of cue, S1, and R1. After completing the first practice, the response rule for the R2 was introduced. In the second practice block (12 trials), the full S1R1-S2R2 task was performed. In each practice block, an accuracy of at least 50% was needed to pass. The accuracy of R1 and R2 responses was combined to evaluate practice block two. If participants missed the criterion in any training session, the experiment ended. Before entering the experiment proper, participants were told to respond as accurately and as fast as possible. Each of the 12 experimental blocks contained 16 trials. After each trial, participants received audio-visual feedback (i.e., the written German words “*richtig!*” [“right!”] or “*falsch!*” [“wrong!”] and corresponding positive or negative spoken words such as “*Ja*” [“yes”], “*Super*” [“awesome”], or “*Nein*” [“no”]) about the correctness of the R1 and R2 responses followed by a 1,200-ms intertrial interval. For R1 responses faster than 100 ms or slower than 1,000 ms and for R2 responses faster than 100 ms or slower than 2,000 ms, respectively, as well as for responses with any key apart from the two response keys, a warning message was presented, and the trial was aborted. A short break was offered after each block, and participants could resume the experiment at their own discretion. During block breaks, a summary of the accuracy of their performance during that block (in percent, for R1 and R2 separately) was presented. In case participants did not continue within 10 min, the experiment was aborted. The experiment took about 50 min to complete.

#### Design and analysis

For all experiments in the series, response times and error rates of R2 (given a correct R1) were of primary interest. Partial repetition costs (PRCs) for response times and error rates were calculated as follows: (Stimulus Change Response Repetition – Stimulus Repetition Response Repetition) – (Stimulus Change Response Change – Stimulus Repetition Response Change)_._ Experiment 1 comprised a 3 × 2 × 2 within-subject design, with RSI (500 ms, 1,500 ms, 5,000 ms), stimulus (repetition vs. change) and response (repetition vs. change) leading to 12 experimental conditions. To assess the after-effect of event file binding and retrieval, the multivariate approach to repeated-measures ANOVA (MANOVA) was applied using Pillai’s trace as criterion in Experiment 1, 3 and 4, more specifically, 3 × 2 × 2 repeated measures MANOVAs were applied to response times and error rates of R2 in Experiment 1. In case of a significant three-way interaction (suggesting the modulation of PRCs by the RSI factor), PRCs were compared between the three levels of the RSI factor (via MANOVA). To fully elucidate the pattern, PRCs were also tested against 0 (via two-sided *t* tests). All *p* values of post hoc comparisons were Bonferroni-Holm corrected.

### Results

#### Data pre-processing

Trials with R1 responses faster than 100 ms (anticipations, 0.3%) and with R1 latencies longer than 1,000 ms (6.7%) were excluded. From the remaining trials, those with probe response latencies shorter than 100 ms (anticipations; 0.0%) or longer than 2,000 ms (0.4%) were additionally excluded from the hypothesis-relevant analyses. No participant was excluded from the final sample due to excessive error rates (40% or more) in R1 or R2 given a valid R1. Out of the 16 trials per cell, on average 15 trials for R1 and 14 trials for R2 were used for response time analysis.

#### Response times

**Fig. 2:**
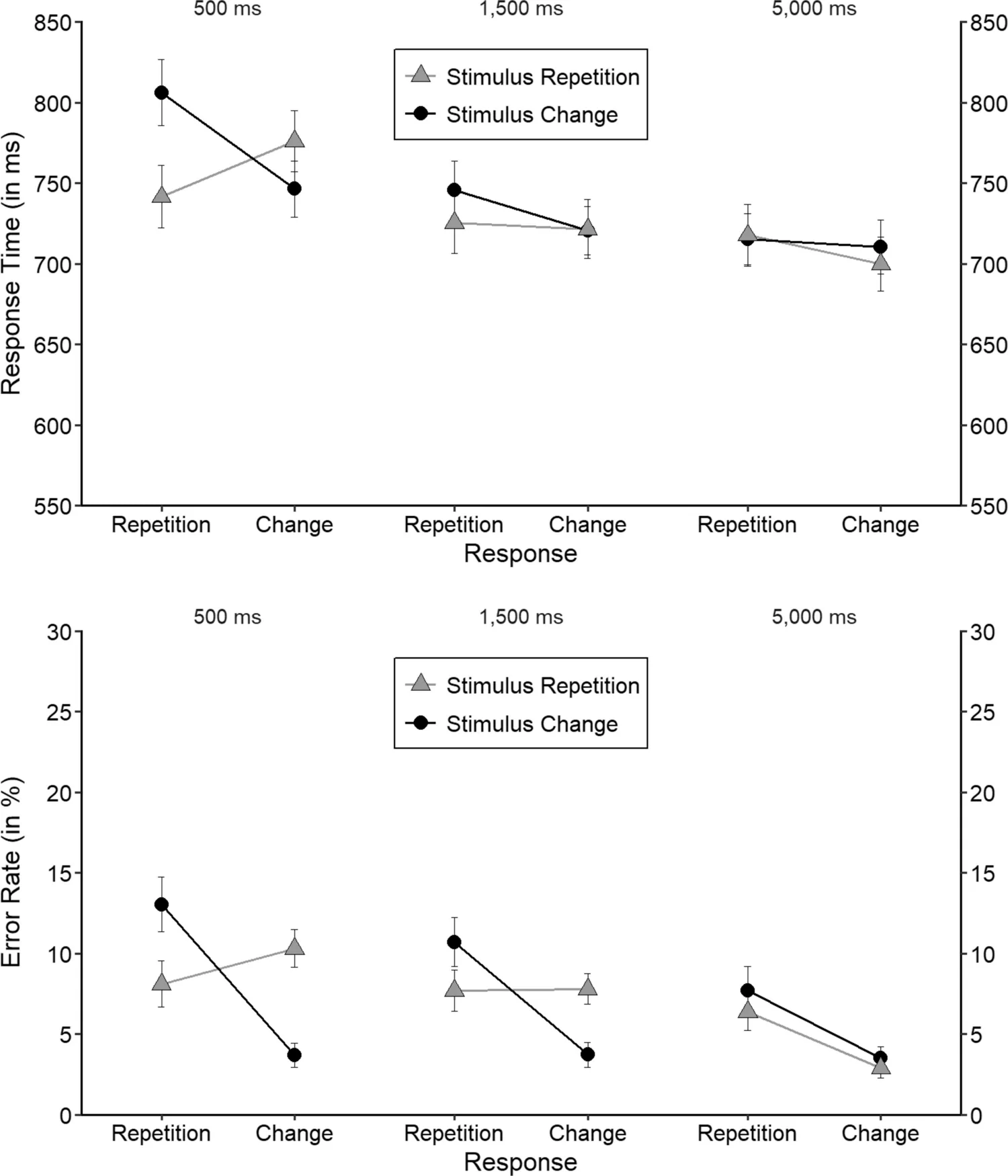
Probe response time and error rate as a function of response, stimulus, and RSI for Experiment 1. *Note.* Error bars represent the standard errors of the means. The upper panel shows response time; the lower panel shows error rate

Main effects of stimulus and response were found, showing that participants responded faster when a stimulus was repeated compared to when it changed, *F*(1, 63) = 5.01, *p* = *.*03, η_p_^2^ = .07, and when a response was changed compared to when it repeated, *F*(1, 63) = 9.29, *p* < *.*01, η_p_^2^ = .13. Response times showed a main effect for RSIs indicating that participants responded faster as RSI increased, *F(*2, 62) = 34.19, *p* < *.*001, η_p_^2^ = .52. S-R binding and retrieval were confirmed by the interaction of stimulus and response, *F*(1, 63) = 19.64, *p* < *.*001, η_p_^2^ = .24, showing longer response times in trials with partial repetitions (i.e., stimulus repetition or response repetition) compared to full or no repetitions (*M*_*PRCs*_ = 34.08 ms, *SD*_*PRCs*_ = 116.18 ms). Most importantly, the interaction of stimulus and response was modulated by the RSIs, *F*(2, 62) = 19.03, *p* < *.*001, η_p_^2^ = .38, see Fig. 2.

**Fig. 3:**
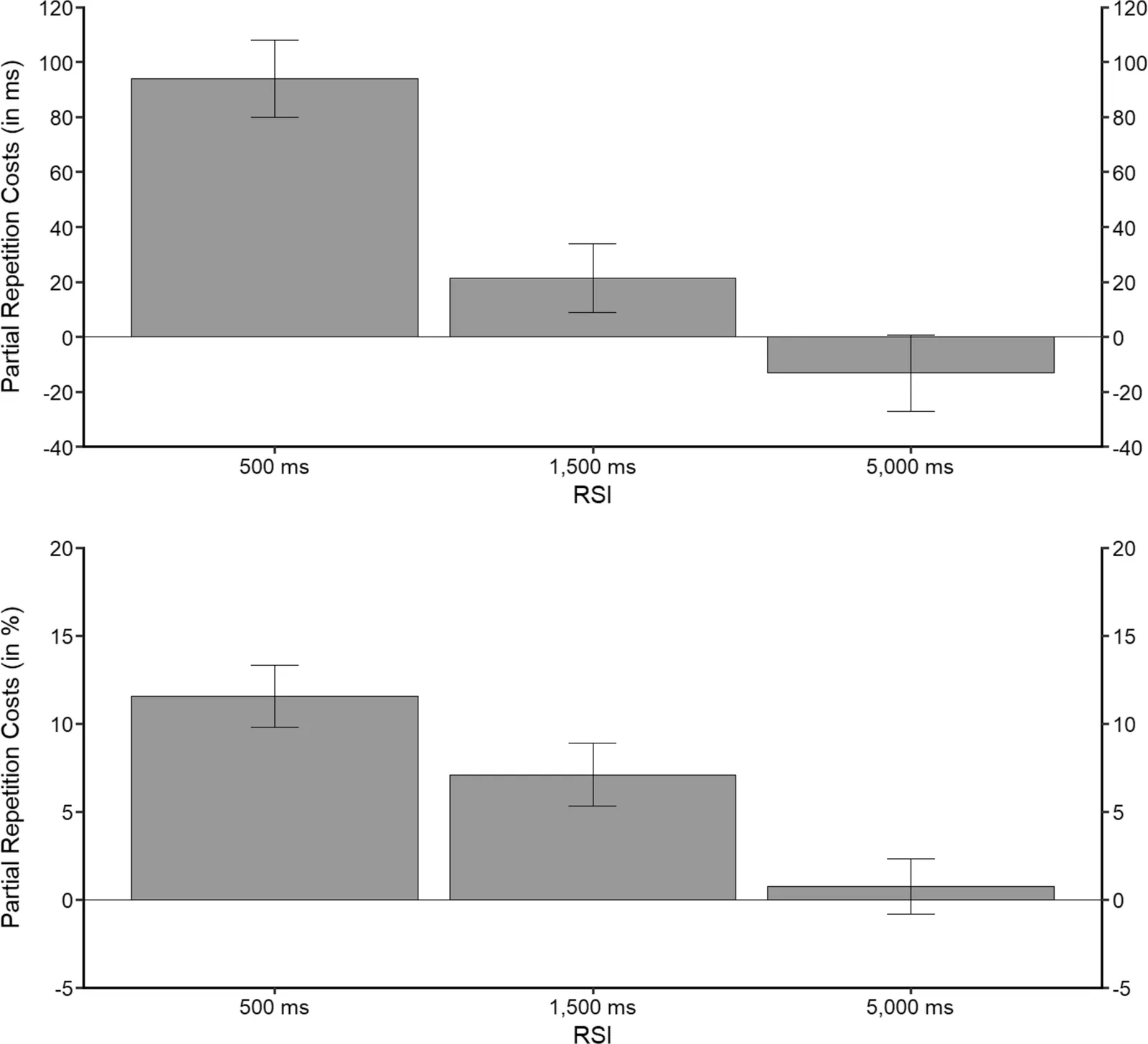
Mean partial repetition costs in milliseconds for probe response time and in percent for probe error rate as a function of RSI for Experiment 1. *Note.* Error bars represent the standard errors of the means. The upper panel shows response time; the lower panel shows error rate

When comparing PRCs among the three levels of the RSI factor, PRCs were larger after the 500 ms as compared with the 5,000 ms RSIs,* F*(1, 63) = 35.38, *p* < *.*001, η_p_^2^ = .36 and after the 500 ms as compared with the 1,500 ms RSIs,* F*(1, 63) = 15.08, *p* < *.*001, η_p_^2^ = .19, however, there was no difference in PRCs between the 1,500 ms and the 5,000 ms RSIs,* F*(1, 63) = 2.79, *p* = .10, η_p_^2^ = .04, visualised in Fig. 3.

Finally, PRCs significantly differed from zero in the 500 ms RSI, *t*(63) = 6.73, *p* < .001, *d*_*z*_ = 0.84, but not after the 1,500 ms and 5,000 ms RSIs with *t*(63) = 1.71, *p* = .19, *d*_*z*_ = 0.21, and *t*(63) = − 0.94, *p* = .35, *d*_*z*_ = -0.12, respectively, visualised in Fig. 3. Descriptive statistics of response times in Experiment 1 are shown in Table 1 in the supplementary material. To keep the results brief, only significant effects are reported. A full overview of the MANOVA results, is provided in Table 2 in the supplementary material.

#### Error rates

There was a main effect of response showing that participants made more errors when the response was repeated compared to when it changed, *F*(1, 63) = 14.34, *p* < *.*001, η_p_^2^ = .19, and a main effect of RSI, showing that more errors were made following short compared to long RSIs, *F*(2, 62) = 16.93, *p* < *.*001, η_p_^2^ = .35. Analogous to results for response times, S-R binding and retrieval were confirmed, as indicated by the interaction of stimulus and response, *F*(1, 63) = 48.36, *p* < *.*001, η_p_^2^ = .43, *M*_*PRCs*_ = 6.5% *SD*_*PRCs*_ = 14.3%. In line with our predictions, the interaction of stimulus and response was modulated by RSI, *F*(2, 62) = 9.22, *p* < *.*001, η_p_^2^ = .23, visualised in Fig. 2.

When comparing PRCs among the three levels of the RSI factor, PRCs were larger after the 500 ms compared to the 5,000 ms RSIs,* F*(1, 63) = 17.87, *p* < *.*001, η_p_^2^ = .22, and after the 1,500 ms compared to the 5,000 ms RSIs,* F*(1, 63) = 7.48, *p* < *.01*, η_p_^2^ = .11, however, there were no significant differences in PRCs between the 500 ms and the 1,500 ms RSIs* F*(1, 63) = 3.03, *p* = .09, η_p_^2^ = .05, visualised in Fig. 3.

Finally, PRCs significantly differed from zero in the 500 ms and the 1,500 ms RSIs with *t*(63) = 6.47, *p* < .001, *d*_*z*_ = 0.81, and *t*(63) = 3.99, *p* < .001, *d*_*z*_ = 0.50, respectively. However, after the 5,000 ms RSI, PRCs disappeared, *t*(63) = 0.48, *p* = .64, *d*_*z*_ = 0.06. Descriptive statistics of error rates in Experiment 1 are shown in Table 3 in the supplementary material. A full overview of the MANOVA results is provided in Table 4 in the supplementary material.

### Discussion

Experiment 1 systematically investigated the effects of time intervals on partial repetition costs (PRCs). If event files are less likely retrieved when separated by a temporal interval, smaller PRCs were expected with long as compared with short intervals. In line with this view, PRCs were generally smaller after medium and long RSIs as compared with short RSIs in response times, and after long intervals compared to medium and short intervals in error rates. Together, the results of Experiment 1 showed smaller PRCs at long RSIs as compared to short RSIs in response times and error rates, consistent with reduced event-file retrieval. This is in line with previous research suggesting that the after-effects of binding vanish at about five seconds (Hommel & Frings, 2020). The reduction of PRCs over increasing intervals has previously been interpreted as evidence that event files disintegrate over time as a function of spontaneous decay (Hommel & Frings, 2020, see General Discussion for a more detailed discourse). Importantly, the results of Experiment 1 represent a conceptual replication of previous findings (Hommel & Frings, 2020), showing that the narrative version of the S1R1 task can be deemed suitable to investigate event-file binding and retrieval. Therefore, the paradigm was employed to investigate whether event file retrieval is also impaired when an event boundary is created by a more abstract representation of time (such as temporal adverbials).

## Experiment 2

Experiment 2^2^ implemented temporal segmentation by means of temporal adverbials (Ezzyat & Davachi, 2011; Speer & Zacks, 2005; Zacks et al., 2007). We were interested whether a temporal adverbial referring to a shorter or longer period, can subjectively segment the sequence of events in a written story very similar to the stories used in Experiment 3 and 4 into units by creating an event boundary. According to EST, an event boundary is more likely to be induced by an adverbial referring to a longer time interval compared to a shorter interval (Ezzyat & Davachi, 2011; Speer & Zacks, 2005; Zacks et al., 2007). The written story in the current study described the trial performance of another person in a narrative version of the S1R1-S2R2 task used in the other experiments of this series. If the mere description of elapsed time affects the perception of an event boundary, participants should perceive this boundary more often after the description of a long as compared with a short time interval. Specifically, the likelihood of experiencing a boundary between the description of the temporal adverbial and the subsequent stimulus (i.e., the S2) should be significantly higher after long (i.e., “after a while”) as compared with short (i.e., “immediately after”) intervals.

### Method

#### Participants

Experiment 2 was pre-registered on aspredicted.org (#243914, https://aspredicted.org/hksw-t3h4.pdf). Power calculations were performed using G*Power 3.1 (Faul et al., 2009). Assuming alpha = beta = .05 and an effect of *d*_*z*_ = 0.40 between the two levels of the temporal adverbial factor, a total sample of 70 was required to achieve a power of .95 in a paired-sample *t* test (one-tailed).

Participants were recruited at the University of Passau and could either enter a lottery to win one of three 30 € Amazon vouchers or receive course credit for their participation. A total of 74 were recruited, but three participants revoked their approval to use their data after completing the study and were excluded from any analyses. The final sample comprised 71 participants with 56 and 15 participants reporting to be female and male, respectively. Age ranged from 19 to 39 years (*M* = 22.7, *SD* = 3.9).

#### Materials and task

In the experiment, participants received written descriptions of trial sequences (i.e., R1 cue, S1, R1, Temporal Adverbial, S2, R2) used in the S1R1-S2R2 task of Experiment 3 and 4. The 2 × 2 × 2 × 2 factorial combination of R1 cue (left vs. right), stimulus (repeated vs. changed), response (repeated vs. changed), and temporal adverbial (short vs. long) resulted in a total of 16 trials for each response assignment for the S2 (i.e., “orange” and “pear” requiring a response on the left- or right-side, respectively). The described response assignment was counterbalanced across participants and trials were presented in random order for each participant. Each trial consisted of the exact wording of stimulus events previously used in Experiment 1 (i.e., “*Die Löwen: links/rechts”* [“The lions: left/right”], “*Eine Orange/Birne erscheint”* [“An orange/pear appears”]), except for the three dots which were replaced with the temporal adverbials (i.e., the German words “*sofort danach*” [ “immediately”] or “*nach langer Zeit”* [“after a while”]), as well as descriptions of the correct responses demanded in the respective trial (i.e., “*Der Abenteurer berührt den Stein auf der linken/rechten Seite”* [“The adventurer touches the stone on the left/right side”]).

**Fig. 4:**
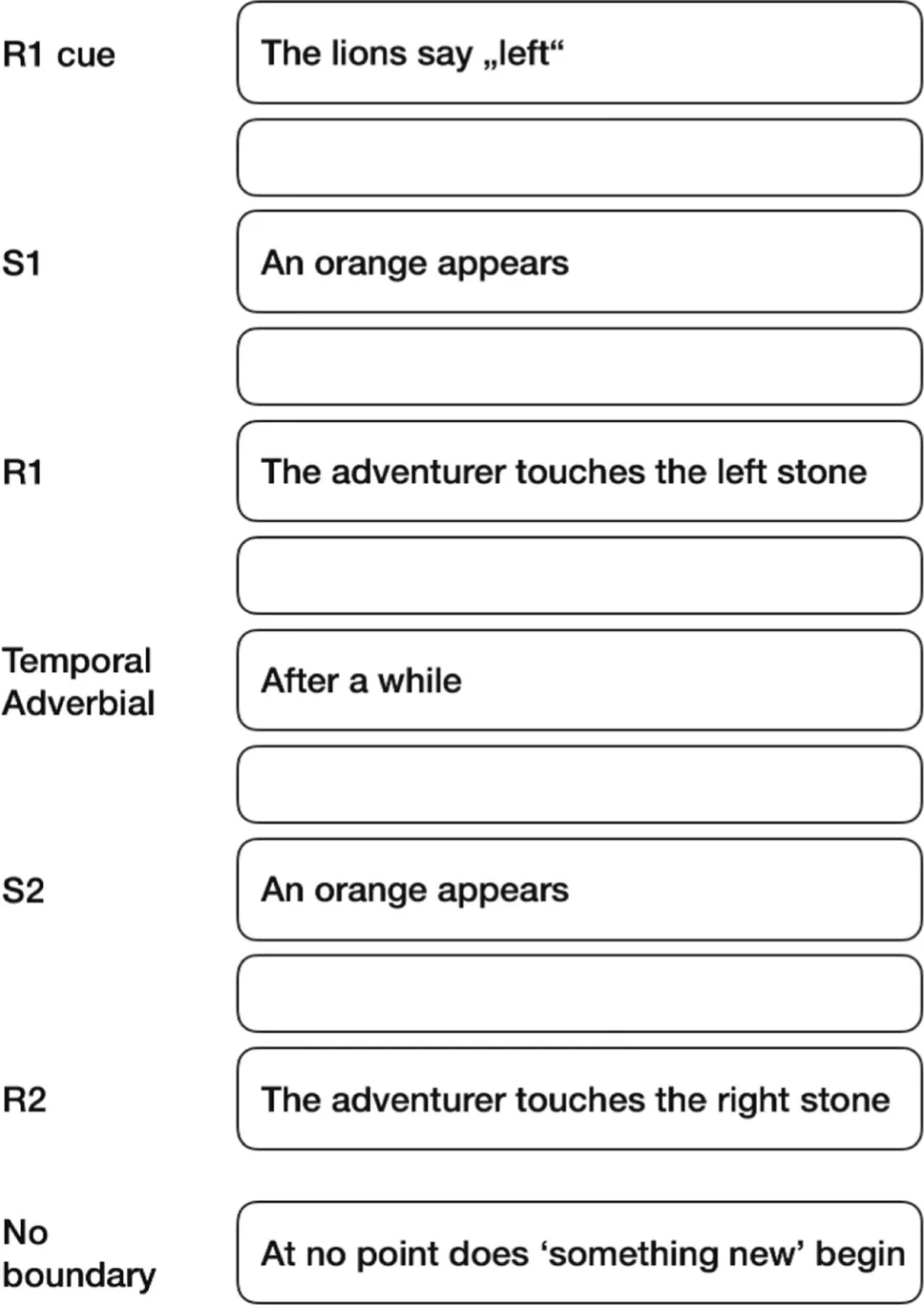
Illustration of a typical trial display for Experiment 2 and its relation to the S1R1-S2R2 task. *Note.* Rounded rectangles represent clickable areas on a computer screen. Relations of each information to the S1R1 paradigm (i.e. R1 cue, S1, R1, S2, R2) are noted on the left of each rectangle. None of the relation descriptions were presented during the experiment. The task required participants to select an empty rectangle via mouse click to determine a boundary between successive written descriptions of events. Clicking a rectangle changed its background colour to signify its selection. Participants were free to modify their choice until a “continue” button (not displayed in the figure) was clicked. Attempting to proceed without selecting an empty rectangle prompted a warning and participants remained in the respective trial until a valid choice was made

Participants were initially told that they would read through a fictional story starring a male or female adventurer taking on the “Trial of the Lions” (German words “*Turnier der Löwen”*). The gender of the adventurer was identical for all trials but counterbalanced across participants. Stimuli and responses in each trial sequence were presented as individual sentences from top to bottom in the order of occurrence in the general task structure of Experiment 1. All sentences appeared at once on the screen but were organised in a line-by-line manner with a clickable area between sentences. In each trial, participants were asked to indicate whether, and if so, where in the written sequence “a meaningful event ends and something new” begins. This was done by selecting the area between two respective sentences where they located the boundary. Alternatively, participants could indicate that they did not perceive a “boundary between events” by selecting the corresponding option (i.e., “*An keiner Stelle beginnt etwas neues”* [ “At no point does ‘something new’ begin”]) via mouse click on the bottom of the page. Participants could only make one decision per trial and all sentences remained visible until a decision was made. Figure 4 illustrates a typical trial display.

#### Procedure

The study was conducted online using the SoSciSurvey platform (Leiner, 2024). After giving informed consent, participants received an introduction to the general task, followed by two thematically unrelated everyday example trials for the segmentation task. The first one was describing the preparation of a fried egg and the second one the assembly of a tent. Both examples were presented in the line-by-line form of the experimental trials. Across all practice and experimental trials, it was highlighted that there was no objectively correct answer and participants were instructed to rely on their intuition when making decisions. The practice trials were followed by a general introduction to the storyline identical to the instruction texts used in Experiment 1. Afterwards, participants worked through 16 experimental trials at their own discretion, followed by two demographic questions about their age and reported gender. Afterwards, participants were informed about the research question and were given the option to submit comments and revoke their consent to use their data. Finally, participants received a participation code that could be redeemed for course credit or entered a lottery to win one of three 30 € Amazon vouchers. On average, the study took 7.41 min (*SD* = 2.67) to complete.

#### Design and analysis

The study comprised a within-subjects design with “Temporal Adverbial” (short vs. long) as the sole hypothesis-relevant factor. The probability of identifying a boundary after the presentation of the temporal adverbial (and before the presentation of S2) was calculated as the number of decisions favouring this section divided by the eight decisions made by each participant for each temporal adverbial factor level. The probability values of the two levels of the temporal adverbial factor were compared by means of a Wilcoxon signed-rank test with continuity correction (one-sided). The exploratory tests are two-sided.

### Results

First, the results from the hypothesis-relevant effect of the temporal adverbial factor on the probability of indicating an event boundary between the description of the adverbial and the S2 will be reported. For exploratory reasons, the probability of experiencing an event boundary between the description of the R1 and the adverbial, as well as reporting no boundary in the narrative descriptions will be compared as a function of the temporal adverbial factor.

**Fig. 5:**
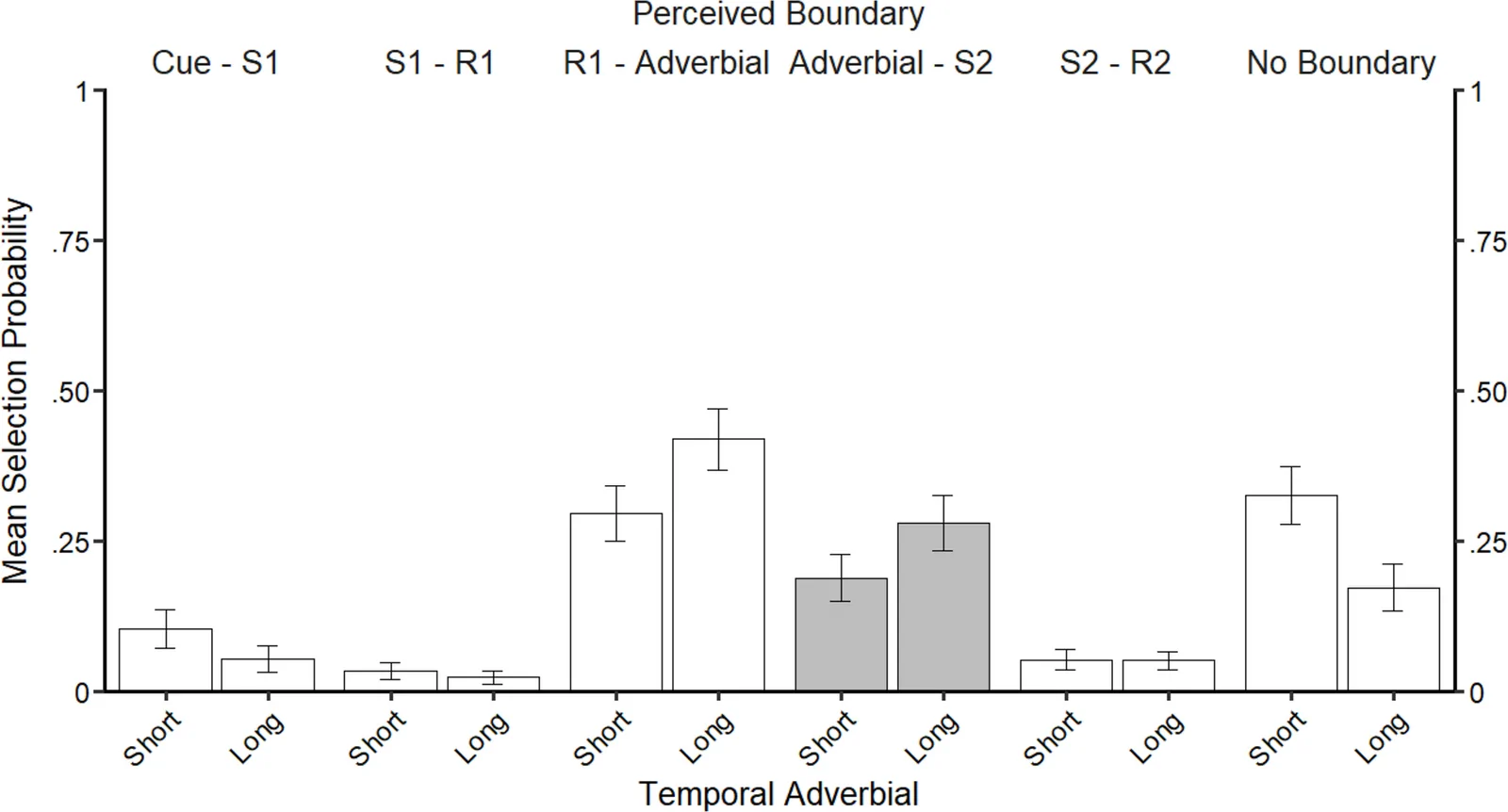
Mean selection probability to perceive an event boundary between each event description as a function of temporal adverbial for Experiment 2. *Note.* The hypothesis-relevant results for the boundary between the temporal adverbial and the S2 are shown in grey. Error bars represent the standard errors of the means

The descriptive results (see Fig. 5 and Table 5 in the supplementary material) showed a larger mean probability to perceive an event boundary after the presentation of a temporal adverbial that referred to a long (*M* = .28, *SD* = .39) as compared with a short interval (*M* = .19, *SD* = .33). Given that the probability differences between the two levels of the temporal adverbial factor significantly deviated from normality (Shapiro–Wilk test with *W*(71) = 0.82, *p* < .001), a Wilcoxon signed-rank test with continuity correction was applied. The test revealed a significant shift in the distribution of probability differences between the long and the short temporal adverbial (*V* = 413.00, *Z* = 1.98, *p* = .024, *r* = .34).

The following analyses were not preregistered and are reported for exploratory purposes only. The descriptive results showed a larger mean probability to perceive an event boundary between the R1 and the temporal adverbial when the adverbial referred to a long (*M* = .42, *SD* = .43) as compared with a short interval (*M* = .30, *SD* = .40). Given that the probability differences between the two levels of the temporal adverbial factor significantly deviated from normality (Shapiro–Wilk test with *W*(71) = 0.89, *p* < .001), a Wilcoxon signed-rank test with continuity correction was applied. The test revealed a significant shift in the distribution of probability differences between the long and the short temporal adverbial (*V* = 535.50, *Z* = 2.03, *p= *.043, *r* = .33). Finally, participants were more likely to perceive no event boundary in the short (*M* = .33, *SD* = .40) as compared with the long (*M* = .17, *SD* = .33) temporal adverbial. Given that the probability differences between the two temporal adverbials significantly deviated from normality (Shapiro–Wilk test with *W*(71) = 0.78, *p* < .001), a Wilcoxon signed-rank test with continuity correction was applied. The test revealed a significant shift in the distribution of probability differences between the long and the short temporal adverbial (*V* = 113.00, *Z* = 3.01, *p* = .002, *r* = .53).

### Discussion

The results of Experiment 2 are straightforward: In line with our predictions, participants were more likely to perceive an event boundary between the description of a temporal adverbial and the description of the following S2 when the adverbial referred to a longer as compared with a shorter time interval. This finding demonstrates that temporal adverbials can effectively guide the segmentation of stories of limited narrative richness and rigid structure as those used in the other experiments in this series by conveying different durations of elapsed time. Supporting this conclusion, exploratory analyses revealed a similar pattern for the potential event boundary between R1 and the temporal adverbial. Again, an event boundary was inferred with a higher probability when a long temporal adverbial followed the R1 than when a short adverbial did. Finally, the temporal adverbial also affected the general tendency to infer a boundary at all. Specifically, participants were more likely to infer no boundary between the described events when the adverbial referred to a shorter as compared with a longer interval. Together, the findings highlight the sensitivity of event segmentation processes to these specific semantic cues referring to time intervals. This sensitivity to symbolic temporal information is consistent with research on temporal orienting, which has shown that linguistic or symbolic cues referring to time can effectively direct attention to anticipated moments in time and thereby modulate behaviour (Nobre, 2001).

## Experiment 3

Experiment 3 investigated whether temporal adverbials referring to short or long periods of elapsed time (i.e., “immediately after” or “after a while”) presented in the fixed time interval between the R1 and the S2 can segment event files. According to EST, an event boundary is more likely to be induced by an adverbial referring to a longer time interval compared to a shorter interval (Ezzyat & Davachi, 2011; Speer & Zacks, 2005; Zacks et al., 2007). Following this idea, it was assumed that the likelihood of S-R retrieval is reduced when prime and probe are separated by a distinct event boundary (in the long adverbial condition), leading to smaller PRCs with an adverbial referring to a long as compared to a short time interval.

### Experimental design and task

**Fig. 6:**
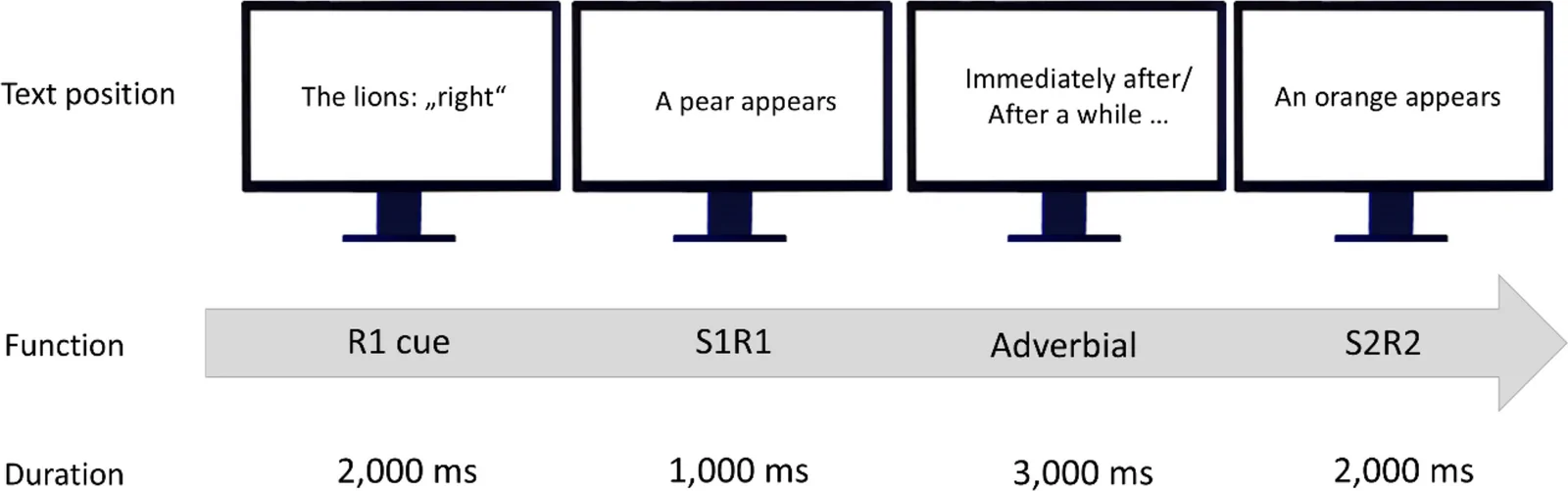
Trial sequence of Experiment 3. *Note*. S1 (S2) refers to the first (second) stimulus, respectively. Responses to the stimuli (R1 for S1 and R2 for S2) are signalled by the cue (R1) or are guided by the response rule (R2). Attention check trials are not depicted

In general, the experiment followed the same procedure as Experiment 1 with the following two exceptions: Only one RSI of 3,000 ms was used and so-called *attention check trials* were randomly presented during each block (12 in total) once after the S1 instead of the adverbials to ensure that adverbials were processed. In those attention check trials, participants were not allowed to perform R2 but had to press the space bar instead at the time of the temporal adverbial presentation. In the context of the narrative story, attention check trials were introduced by describing that occasionally, the lions were “silent” (presentation of the German words “*Die Löwen bleiben stumm”* [“the lions remained silent”]) at the time of the temporal adverbial; the S2 and R2 of the trial were removed. Those trials were practiced individually in a third practice block (six trials). With the addition of attention check trials, each of the 12 blocks contained 17 trials leading to a total of 204 trials with 24 trials per cell. The experiment took about 45 min to complete. A breakdown of the trial sequence is shown in Fig. 6.

### Method

#### Participants

Experiment 3 was pre-registered on aspredicted.org (#155688, https://aspredicted.org/4834-sgd6.pdf). Statistical power analysis followed the same rationale as in Experiment 1, but the temporal adverbial only had two levels instead of three and thus data had to be collected from 75 participants (assuming a medium-sized modulation effect of PRCs by temporal adverbial of* f* = 0.25, alpha = beta = .05 and a correlation of ρ = .3 between the measures of the PRCs). Seventy-four participants were recruited. Two participants with excessive overall error rates of 40% or more in R1 were excluded from the statistical analysis, both also had an error rate of more than 40% in R2. Two additional participants were excluded due to an error rate of 50% or more for the attention check trials^3^ . Thus, the final sample comprised 70 participants (38 women, 32 men) and an age range from 18 to 39 years (*M* = 25.2, *SD* = 5.2). With the final sample size, power (.94) was only marginally lower than originally planned for. However, it should be noted that the target sample size of 75 was not fully met after exclusions. This should be kept in mind when interpreting the null results of Experiment 3, as the reduced sample could have limited the ability to detect small adverbial effects if they existed. The Bayes factors reported below provide additional information about the strength of the null results independently of the sample size.

#### Design and analysis

Experiment 3 comprised a 2 × 2 × 2 within-subject design, with temporal adverbial (short vs. long), stimulus (repetition vs. change) and response (repetition vs. change) leading to 8 experimental conditions. 2 × 2 × 2 repeated measures MANOVAs were applied to response times and error rates of Experiment 3. To evaluate null effects, Bayes factors were computed with JASP (JASP-Team, 2025).

### Results

#### Pre-processing

Trials were discarded if R1 responses were faster than 100 ms (anticipations, 0.3%) or longer than 1,000 ms (5.2%). From the remaining trials, those with R2 responses faster than 100 ms (anticipations, 0.0%) or longer than 2,000 ms (0.6%) were additionally discarded from the hypothesis-relevant analyses. Mean error rate in attention check trials was *M* = 11.55% (*SD* = 11.20%), mean response time in attention check trials was* M* = 1182.7 ms (*SD* = 240.6 ms). Out of the 24 trials per cell, on average 23 trials for R1 and 21 trials for R2 were used for response time analysis.

#### Response times

**Fig. 7:**
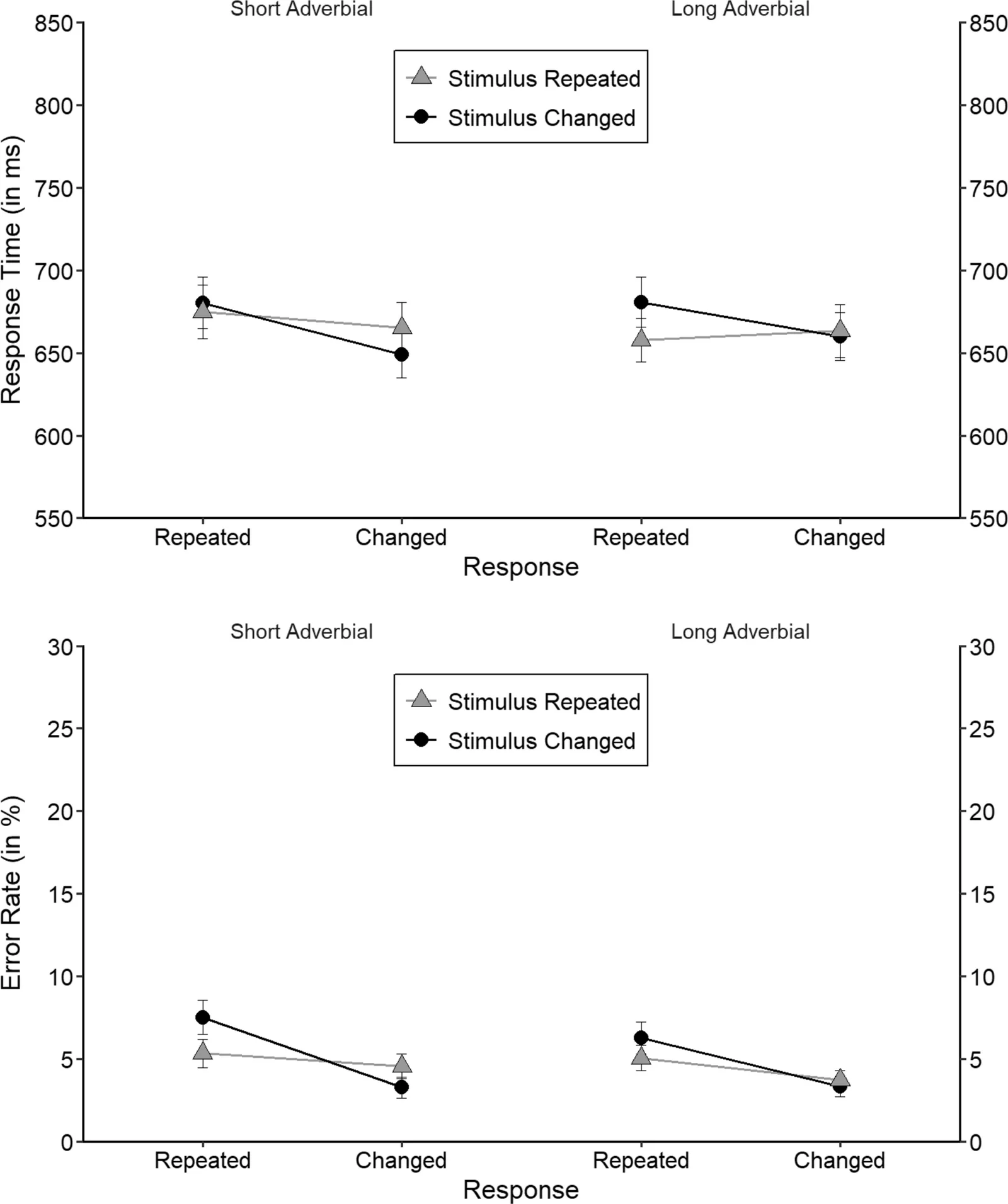
Probe response time and error rate as a function of response, stimulus, and temporal adverbial for Experiment 3. *Note.* Error bars represent the standard errors of the means. The upper panel shows response time; the lower panel shows error rate

**Fig. 8:**
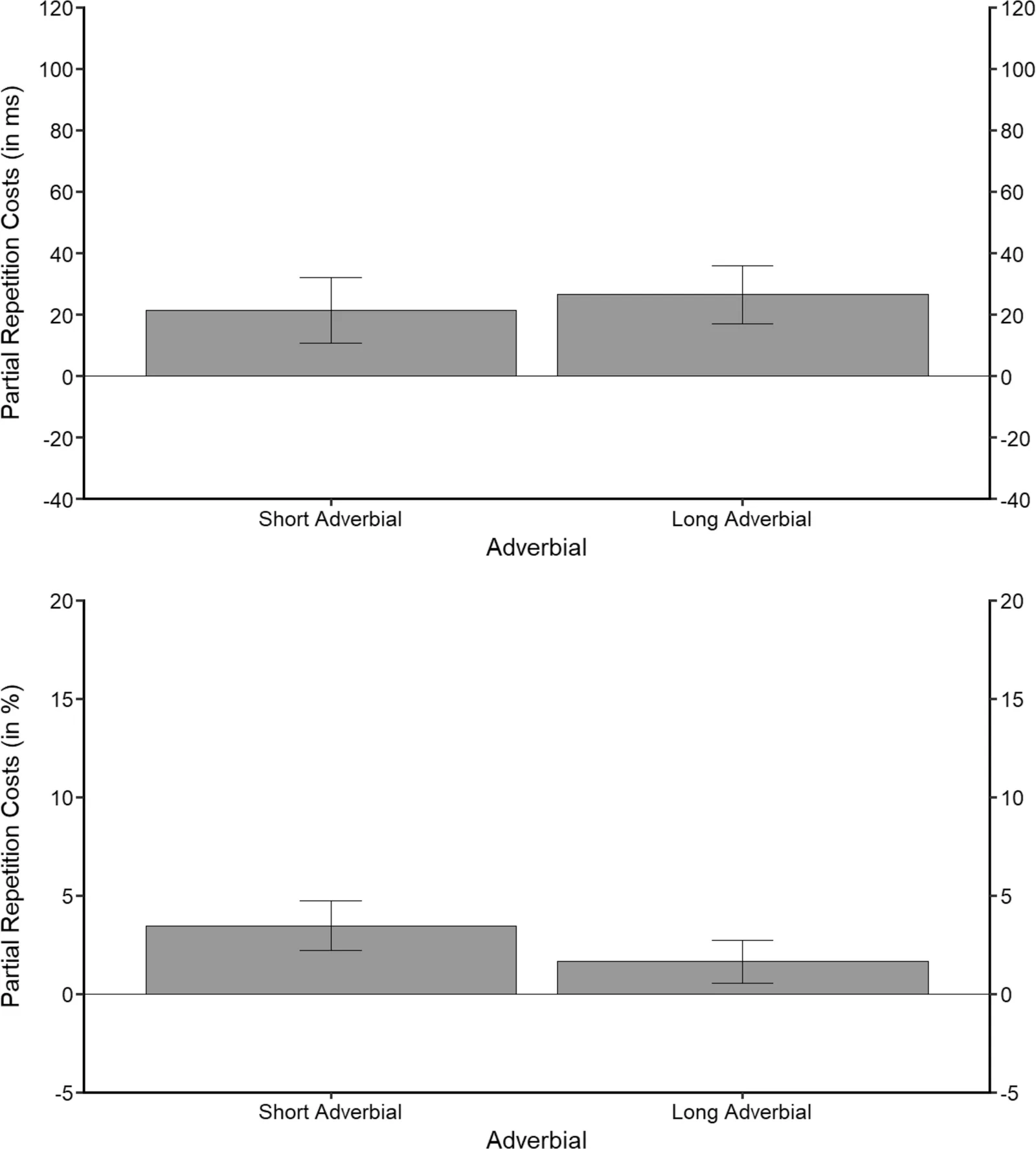
Mean partial repetition costs in milliseconds for probe response time and in percent for probe error rate as a function of temporal adverbial for Experiment 3. *Note.* Error bars represent the standard errors of the means. The upper panel shows response time; the lower panel shows error rate

Participants were faster when the response changed compared to when it repeated, which is visible in a main effect of response, *F*(1, 69) = 18.70, *p* < *.*001, η_p_^2^ = .21. This effect was stronger for the adverbial referring to a short period than the adverbial referring to a long period, as indicated by an interaction of response and adverbial, *F*(1, 69) = 4.30, *p* = *.*04, η_p_^2^ = .06. The interaction of stimulus and adverbial shows that response times were also shorter when the stimulus changed compared to when it repeated for the short adverbial but response times were longer when the stimulus changed to when it repeated for the long adverbial, *F*(1, 69) = 6.70, *p* = *.*01, η_p_^2^ = .09. Results confirmed S-R binding and retrieval as indicated by the interaction of stimulus and response, *F*(1, 69) = 8.91, *p* < *.*01, η_p_^2^ = .11, with slower responses in partial repetition trials compared to full or no repetitions (*M*_*PRCs*_ = 23.88 ms *SD*_*PRCs*_ = 83.87 ms). However, retrieval was not modulated by the temporal adverbial: no significant three-way interaction of stimulus, response, and adverbial, *F*(1, 69) = 0.18, *p* = .67, η_p_^2^ < .01, *BF*_01_ = 2.45, see Figs. 7 and 8 for PRCs. For descriptive statistics of Experiment 3, please see Table 6 in the supplementary material. A full overview of the MANOVA results is provided in Table 7 in the supplementary material.

#### Error rates

More errors were made when the response repeated compared to when the response changed, which can be seen in the main effect of response, *F*(1, 69) = 19.40, *p* < *.*001, η_p_^2^ = .22. S-R binding and retrieval could also be confirmed in error rates as indicated by the interaction of stimulus and response, *F*(1, 69) = 10.28, *p* < *.*01, η_p_^2^ = .13, with more errors in partial repetition trials compared to full or no repetitions (*M*_*PRCs*_ = 2.5% *SD*_*PRCs*_ = 9.9%). Again, retrieval was not modulated by the adverbials in error rates (no significant three-way interaction of stimulus, response, and adverbial, *F*(1, 69) = 1.08, *p* = *.*30, η_p_^2^ = .02, *BF*_01_ = 2.36), see Figs. 7 and 8 for PRCs. For descriptive statistics of Experiment 3, please see Table 8 in the supplementary material. A full overview of the MANOVA results is provided in Table 9 in the supplementary material.

### Discussion

Experiment 3 systematically investigated the effects of mental representations of time (operationalized by temporal adverbials) on PRCs in the probe. Smaller PRCs, interpreted as reflecting a reduced likelihood of event-file retrieval, were expected with temporal adverbials referring to longer (as compared with shorter) periods of time. While we found significant after-effects of event file binding and retrieval, the results of Experiment 3 revealed no segmenting effect of temporal adverbials on PRCs, providing no evidence for a modulation of event-file retrieval by temporal adverbials. One of two conclusions might be drawn from the current results: First, it might be that temporal adverbials were not (sufficiently) processed in the current study and were thereby ineffective in their ability to modulate event file retrieval. Second, even though temporal adverbials can create an event boundary shown in previous research (Ezzyat & Davachi, 2011) and in the current Experiment 2, the repetitive task sequence and/or the stimuli might have prevented effects from being found.

The first assumption seems unlikely because the implementation of attention check trials required participants to process the adverbial information in the current task setting. Given the correct response rate in the attention check trials (88.5%), it seems plausible to assume that the temporal adverbials were read. Moreover, it can be highlighted that for the response time measure, the temporal adverbial factor entered into significant two-way interactions with both the stimulus factor and the response factor. To reiterate, response times were shorter when the stimulus changed compared to when it repeated for the short adverbial, but response times were longer when the stimulus changed to when it repeated for the long adverbial. Moreover, responses were faster in response change compared to response repeated trials, and the effect was stronger for the adverbial referring to a short period than the adverbial referring to a long period. These two-way interactions can be seen as evidence that the adverbials were processed and exerted some influence on the effect of stimulus and response factors.

The second statement suggests that our manipulation might not be sufficient to create an event boundary that affects the likelihood of retrieval of an event file. Both adverbials were presented for the same duration (i.e., 3,000 ms) in Experiment 3, so that, while being semantically related to durations, the *actual* time that elapsed was always the same. It could also be argued that the adverbials were presented as part of a repetitive trial sequence, in which the same sentences with few variations were used throughout the experiment. Under such conditions, participants may have processed the adverbials in a rather shallow manner. In other words, while participants might have read the adverbials, they might not have necessarily captured the semantic meaning of the adverbials and, moreover, might not have integrated the meaning into the context of the overall narrative story. This assumption would be consistent with the absence of adverbial effects on PRCs. However, attention check trial performance confirms that participants attended the adverbials and in addition to that, the adverbials entered two interactions with the stimulus and the response factor which shows that participants have differentiated between the two levels of the adverbial factor requiring semantic processing of the words.

Another consideration is that the fixed RSI of 3,000 ms in Experiment 3 may itself have attenuated PRCs to a level that left little room for further modulation by the adverbial. The overall PRCs observed in Experiment 3 were smaller than those found at the 500 ms RSI in Experiment 1, and PRCs at the 1,500 ms RSI in Experiment 1 did not significantly differ from zero, suggesting that much of the decay in trace strength may already have occurred by 3,000 ms. If veridical time and symbolic temporal information exert largely independent influences on trace strength, the relatively long fixed interval may have reduced PRCs to a level at which detecting any additional adverbial effect was difficult. Whether strengthening the association between the adverbials and actual temporal distance could promote deeper processing of their meaning was investigated in Experiment 4.

## Experiment 4

Considering that the combined results from Experiment 1 suggest that time intervals can reduce event file binding and retrieval, whereas temporal adverbials cannot (or at least did not in Experiment 3), we tested whether we could enhance the perception of temporal distance indicated by the adverbials by means of actual temporal distance. By pairing temporal adverbials with average interval length, we aimed at strengthening the processing of the adverbial manipulation. To this end, each of the two temporal adverbials used in Experiment 3 was varied systematically with three RSI levels. Importantly, the intervals associated with temporal adverbial referring to the short interval were on average shorter than the intervals associated with temporal adverbials referring to the long interval. More precisely, the “immediately after” adverbials were presented for either 500 ms, 2,000 ms, or 3,000 ms, while the “after a while” adverbials were presented for 3,000 ms, 4,000 ms, or 5,500 ms. To solely assess the effect of the adverbial manipulation only the 3,000 ms RSIs were analysed. The remaining two intervals in each adverbial condition (i.e., 500 ms and 2,000 ms, as well as 4,000 ms and 5,500 ms, respectively) were only employed to manipulate the average time interval associated with each adverbial. Trials including intervals shorter or longer than 3,000 ms were not included in the hypothesis-relevant main statistical analyses, but nevertheless, effects of time intervals were analysed exploratorily. Analogous to the previous hypotheses, we expected that an event boundary is more likely to be induced by an adverbial referring to a longer time interval compared to a shorter interval (Ezzyat & Davachi, 2011; Speer & Zacks, 2005; Zacks et al., 2007). Following this notion, we assumed that event files are less likely to be retrieved when they are separated by a distinct event boundary (i.e., in the long adverbial condition), leading to smaller PRCs with an adverbial referring to a longer as compared with a shorter interval.

### Experimental design and task

**Fig. 9:**
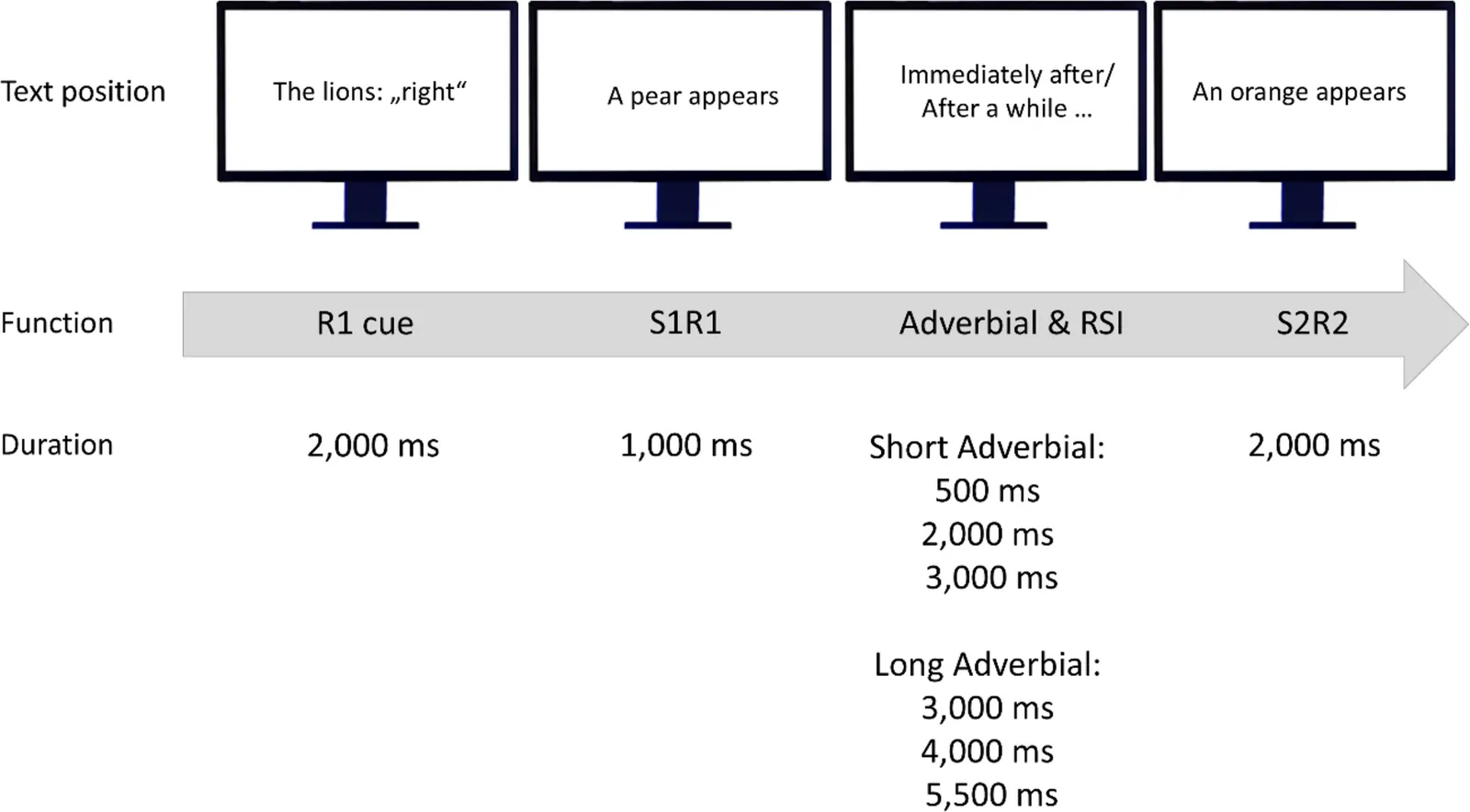
Trial sequence of Experiment 4. *Note*. S1 (S2) refers to the first (second) stimulus, respectively. Responses to the stimuli (R1 for S1 and R2 for S2) are signalled by the cue (R1) or are guided by the response rule (R2). Memory checks are not depicted

The design and task used in Experiment 4 was identical to Experiment 3 with the following exception. RSI was varied at three levels — short (500 ms and 2,000 ms, paired with the short adverbial only), medium (3,000 ms, paired with both adverbials), and long (4,000 ms and 5,500 ms, paired with the long adverbial only) — to strengthen the segmenting effect of the temporal adverbial, since the mean RSI associated with the short temporal adverbial (500 ms, 2,000 ms, 3,000 ms) was shorter than for the long one (3,000 ms, 4,000 ms, 5,500 ms). The experiment included 256 trials, with 16 trials per block (16 blocks in total). Trials with a duration of 3,000 ms made up 50% of all trials (128), out of those, 64 trials were combined with the short adverbial, and 64 trials were combined with the long adverbial resulting in 32 trials per cell. All other intervals occurred 32 times; the 500 ms and 2,000 ms intervals were paired with the short adverbial; the 4,000 ms and 5,500 ms intervals were paired with the long adverbial only. For the hypothesis-relevant statistical analyses, only trials of the same duration were considered (3,000 ms). For exploratory analyses of the RSIs, all trials except 3,000 ms were considered as these were combined with both adverbials. As attention check trials in Experiment 1 led to the exclusion of trials, four memory checks for the temporal adverbials were included in each block in Experiment 4 to ensure that temporal adverbials were processed. For those, the participants performed a forced choice binary memory check task without time limit (German words “*Im vorangegangenen Durchgang wurde eine Zeitangabe gemacht. Was war die Zeitangabe?”* [“In the previous round, a time reference was given. What was that time?”]) and answered via a mouse click on the button labelled “*Sofort danach”* [immediately after”] or “*Nach langer Zeit”* [“after a while”]. For a breakdown of the trial sequence, see Fig. 9.

### Method

#### Participants

The experiment was pre-registered on aspredicted.org (#171750, https://aspredicted.org/r8kg-mksp.pdf). Power analysis analogous to the second experiment determined that data had to be collected from 75 participants (assuming a medium-sized modulation effect of PRCs by temporal adverbial of f = 0.25, alpha = 1-beta = .05 and a correlation of ρ = .3 between the measures of the PRCs). Seventy-nine participants were recruited, but five participants had to be excluded due to excessive error rates in memory checks for the temporal adverbial (40% or more of responses were incorrect,^4^). No one was excluded due to excessive error rates in R1 or R2 given a valid R1. Seventy-four (53 women, 19 men, 2 nonbinary) participants comprised the final sample, and age ranged from 19 to 36 years (*M* = 22.8, *SD* = 3.2).

#### Design and analysis

The experiment comprised a 2 × 2 × 2 × 3 within-subject design, with temporal adverbial (short vs. long), stimulus (repetition vs. change) response (repetition vs. change), and RSI (short: 500 ms and 2,000 ms, paired with the short adverbial only; medium: 3,000 ms, paired with both adverbials; long: 4,000 ms and 5,500 ms, paired with the long adverbial only) as independent variables, leading to 24 experimental conditions. A 2 × 2 × 2 repeated measures MANOVA with the factors temporal adverbial, stimulus, and response was applied to response times and error rates of R2 to all trials with an RSI of 3,000 ms. Additionally, 4 × 2 × 2 repeated measures MANOVAs with the factors RSI, stimulus, and response were applied to response times and error rates of R2 in trials with an RSI of 500 ms, 2,000 ms, 4,000 ms, and 5,500 ms (thus excluding RSI = 3,000 ms) to analyse effects of the RSIs on the after-effects of event file binding in an exploratory manner and PRCs were tested against zero (via two-sided t-tests).

### Results

#### Pre-processing

Trials were discarded if prime responses were faster than 100 ms (anticipations, 0.3%), or longer than 1,000 ms (5.8%). From the remaining trials, those with probe responses faster than 100 ms (anticipations, < 0.1%) or longer than 2,000 ms (0.2%) were additionally discarded from the hypothesis-relevant analyses. Out of the 32 trials per cell, on average 30 trials for R1 and 28 trials for R2 were used for response time analysis. Mean recall accuracy for memory check was M = 84.3% SD = 11.2%.

#### Temporal adverbials

##### Response times

**Fig. 10:**
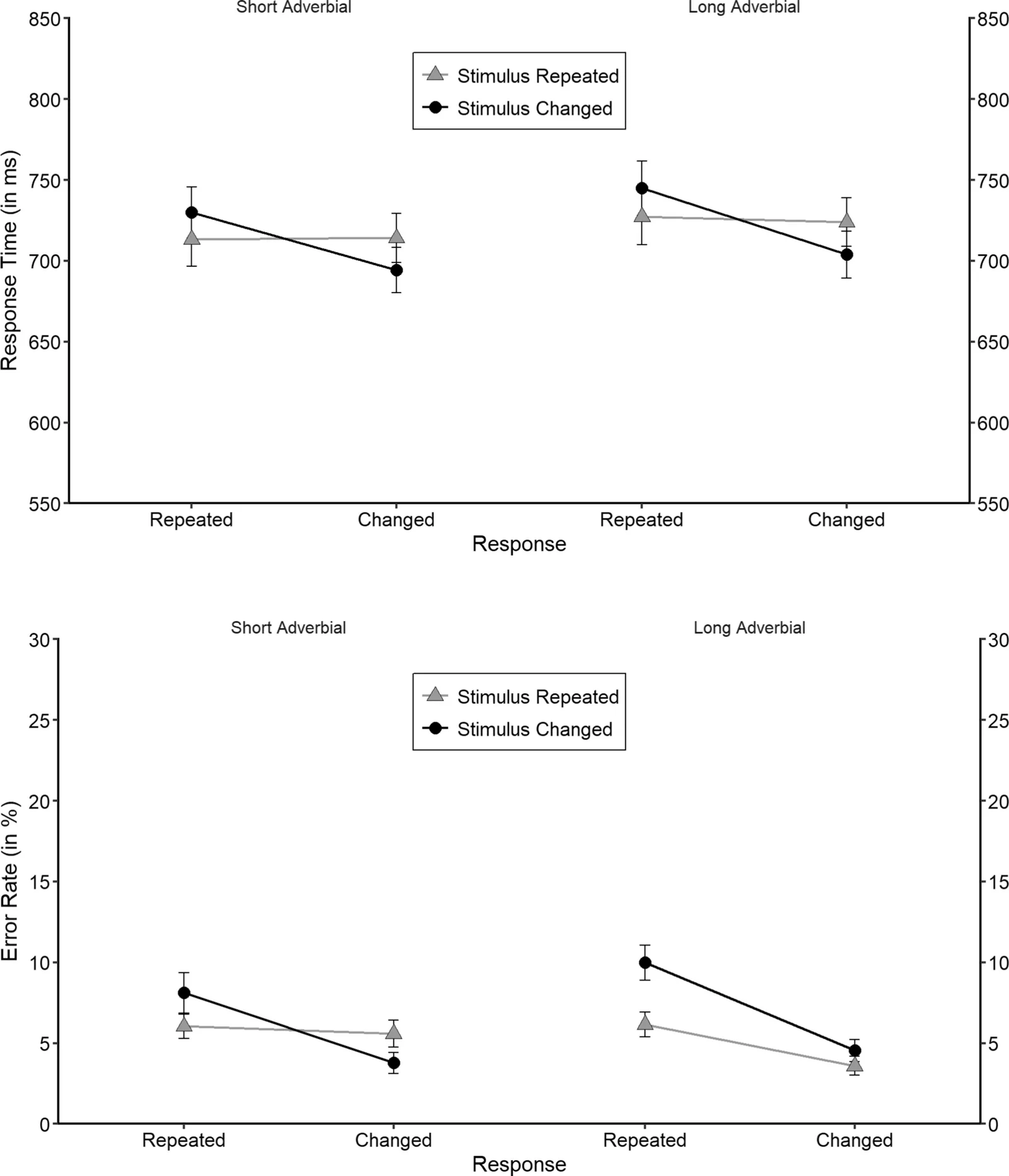
Probe response time and error rate as a function of response, stimulus, and temporal adverbial for Experiment 4. *Note.* Error bars represent the standard errors of the means. The upper panel shows response time; the lower panel shows error rate

**Fig. 11:**
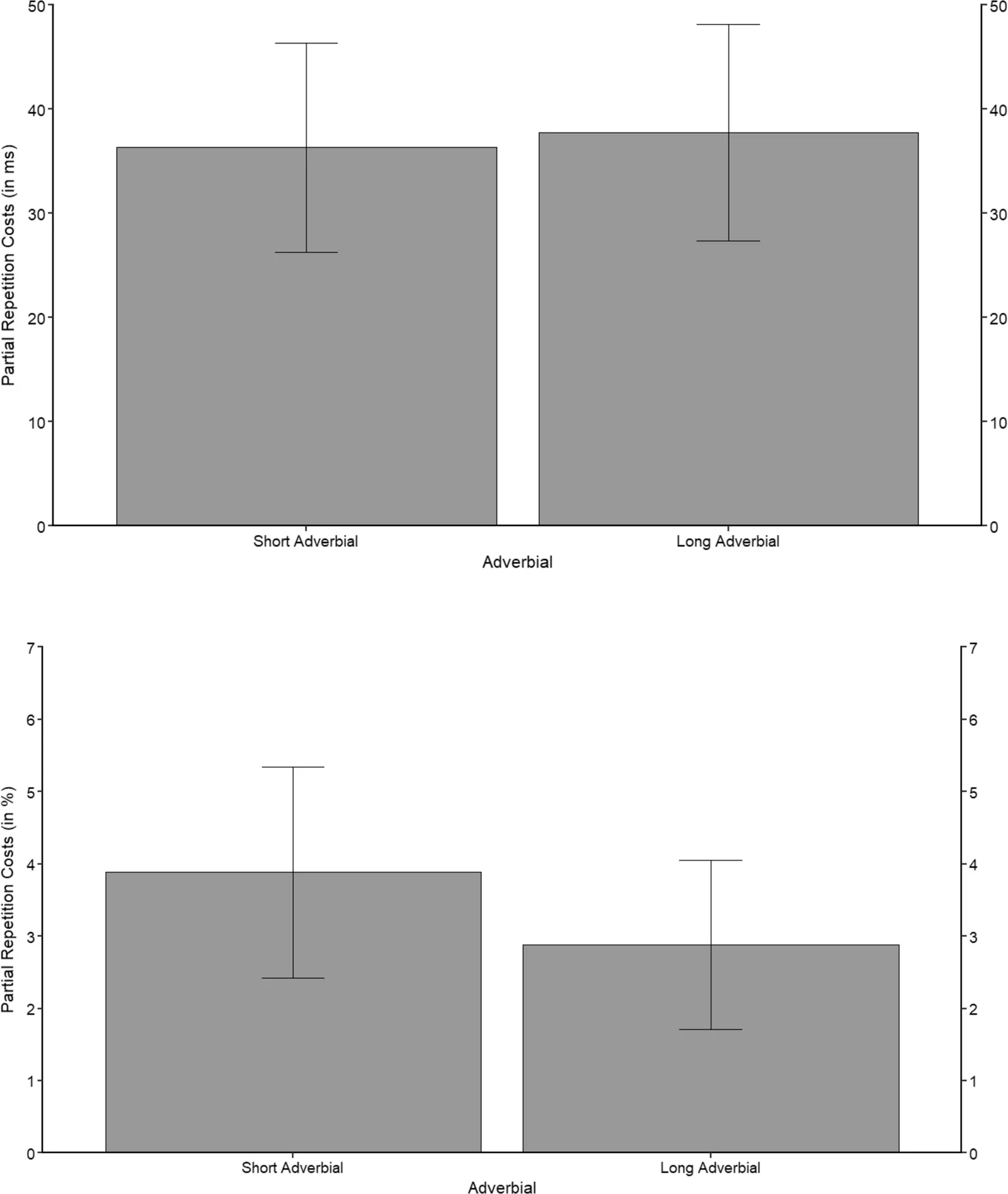
Mean partial repetition costs in milliseconds for probe response time and in percent for probe error rate as a function of temporal adverbial for Experiment 4. *Note.* Error bars represent the standard errors of the means. The upper panel shows response time; the lower panel shows error rate

The 2 × 2 × 2 MANOVA with the factors temporal adverbial, stimulus, and response revealed a main effect of response showing that participants responded faster when a response was changed compared to when a response was repeated, *F*(1, 73) = 18.58, *p* < *.*001, η_p_^2^ = .20. Response times were longer after the adverbials referring to a longer period of time as compared to adverbials referring to a shorter period of time, as visible in the main effect of adverbial, *F*(1, 73) = 7.85, *p* < *.*01, η_p_^2^ = .10, see Fig. 10. There was evidence for S-R binding and retrieval, as indicated by the interaction of stimulus and response, *F*(1,73) = 27.06, *p* < *.*001, η_p_^2^ = .27, *M*_*PRCs*_ = 37.07 ms, *SD*_*PRCs*_ = 87.6 ms. S-R binding and retrieval were not modulated by the adverbial (no significant three-way interaction stimulus, response, and adverbial, *F*(1,73) = 0.01, *p* = .92, η_p_^2^ < .01, *BF*_01_ = 7.08) (see Figs. 10 and 11 for PRCs). For descriptive statistics of Experiment 4, please see Table 10 in the supplementary material. A full overview of the MANOVA results is provided in Table 11 in the supplementary material.

##### Error rates

The main effect of response highlights that more errors were made when the response was repeated compared to when the response changed, *F*(1, 73) = 21.40, *p* < *.*001, η_p_^2^ = .23. Participants made more errors when a stimulus changed compared to when a stimulus repeated, which can be seen in the main effect of stimulus, *F*(1, 73) = 10.33, *p* < *.*01, η_p_^2^ = .12. This was particularly visible for the adverbial indicating a long period; however, less so for the adverbial indicating a short period, as indicated by the interaction effect of stimulus and adverbial, *F*(1, 73) = 6.15, *p* = *.*02, η_p_^2^ = .08. S-R binding and retrieval could also be confirmed, as indicated by the interaction of stimulus and response, *F*(1, 73) = 12.87, *p* < *.*001, η_p_^2^ = .15, *M*_*PRCs*_ = 3.38% *SD*_*PRCs*_ = 11.3%, see Fig. 10. Again, retrieval was not modulated by the adverbials in error rates (no significant three-way interaction of stimulus, response, and adverbial, *F*(1, 73) = 0.29, *p* = *.*59, η_p_^2^ < .01, *BF*_01_ = 4.08, see Figs. 10 and 11 for PRCs). For descriptive statistics of Experiment 4, please see Table 12 in the supplementary material. A full overview of the MANOVA results is provided in Table 13 in the supplementary material.

### Exploratory analysis: Time intervals

#### Response times

**Fig. 12:**
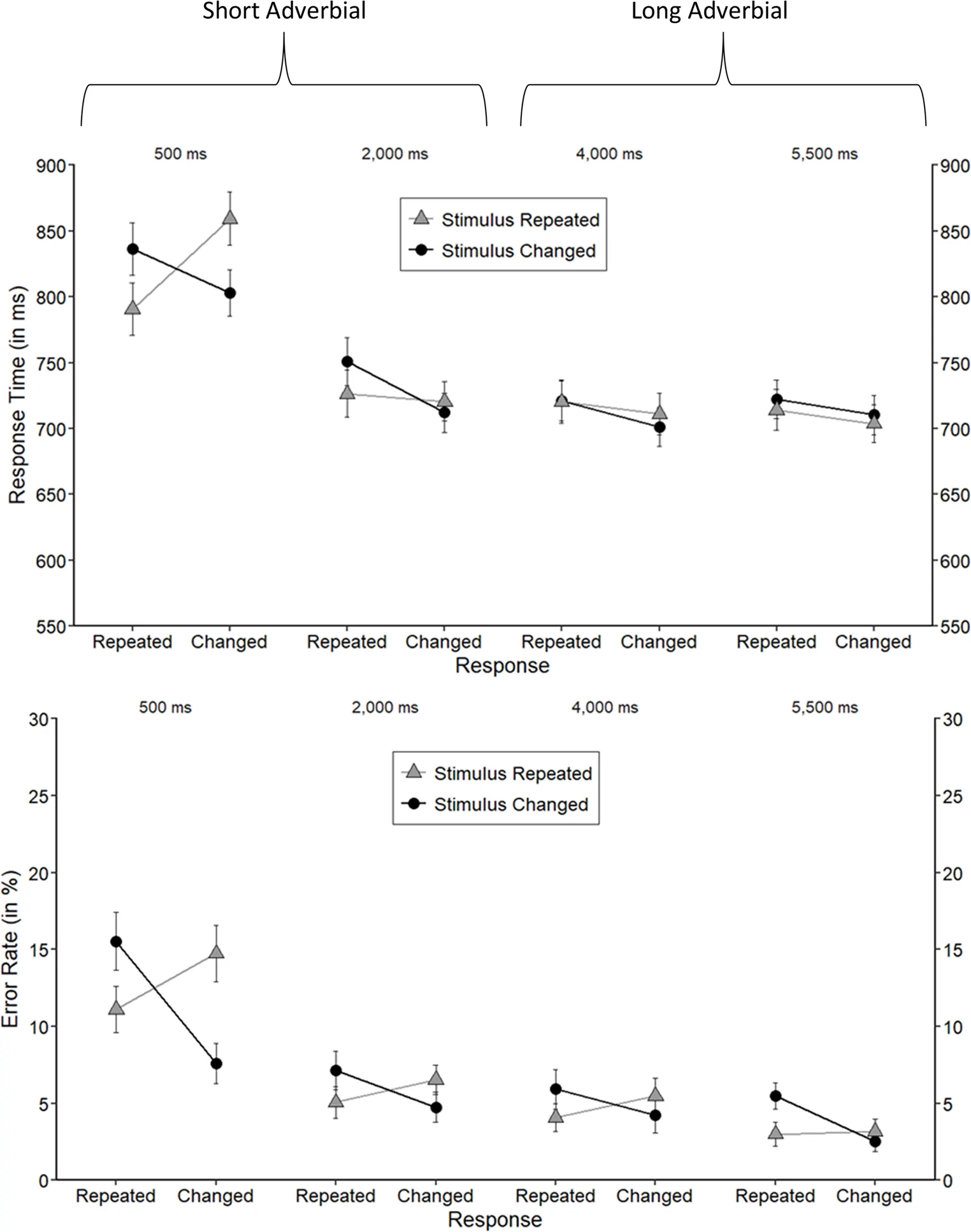
Probe response time and error rate as a function of response, stimulus, RSI, and temporal adverbial for Experiment 4. *Note.* Error bars represent the standard errors of the means. The dashed lines frame the response stimulus interval conditions between R1 and S2 (i.e., 3,000 ms) that were employed in both adverbial conditions. The upper panel shows response time; the lower panel shows error rate

For trials with 500 ms, 2,000 ms, 4,000 ms, and 5,500 ms, response times were longer after the short RSI compared to long RSIs, which is confirmed in the main effect of RSI, *F(*3, 71) = 34.92 *p* < *.*001, η_p_^2^ = .60. For the 500 ms RSI, response times were shorter when the response was repeated compared to when the response changed. However, for the RSIs 2,000 ms, 4,000 ms, and 5,500 ms response times were longer when the response repeated compared to when the response changed, as indicated by the interaction of response and RSI, *F*(3, 71) = 5.00 *p* < *.*01, η_p_^2^ = .17. S-R binding and retrieval were confirmed, as indicated by the interaction of stimulus and response, *F*(1, 73) = 16.66, *p* < *.*001, η_p_^2^ = .19, *M*_*PRCs*_ = 36.87 ms *SD*_*PRCs*_ = 154.57 ms, and additionally, retrieval was modulated by the RSIs (three-way interaction stimulus, response and RSI, *F*(3, 71) = 4.83 *p* < *.*01, η_p_^2^ = .17), as visualised in Fig. 12.

**Fig. 13:**
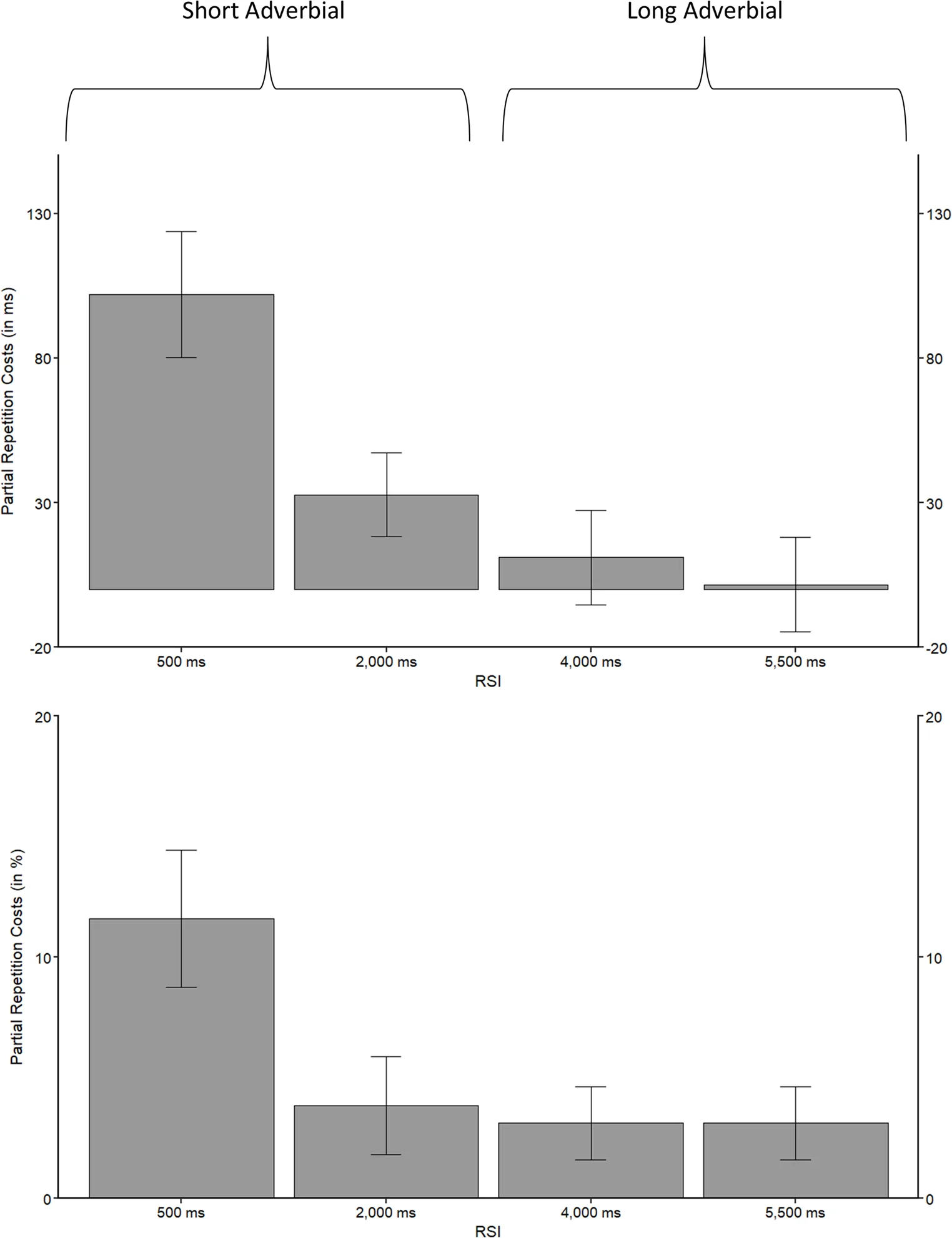
Mean partial repetition costs in milliseconds for probe response time and in percent for probe error rate as a function of RSI and temporal adverbial for Experiment 4. *Note.* Error bars represent the standard errors of the means. The abbreviation “RSI” refers to the response stimulus interval between R1 and S2. The dashed lines frame the RSI conditions (i.e., 3,000 ms) that were employed in both adverbial conditions. The upper panel shows response time; the lower panel shows error rate

PRCs significantly differed from zero in the 500 ms RSI (*t*(73) = 4.69, *p* < .001, *d*_*z*_ = 0.54) and showed a trend toward significance at the 2,000 ms RSI (*t*(73) = 2.27, *p* = .08, *d*z = 0.26), suggesting a gradual decline in PRCs as a function of RSI. At the 4,000 ms and 5,500 ms RSIs, PRCs no longer approached significance (for the 4,000 ms RSI: *t*(73) = 0.67, *p* = 1.00, *d*z = 0.08; for the 5,500 ms RSI: *t*(73) = 0.10, *p* = 1.00, *d*z = 0.01). The three-way interaction indicates that PRCs decreased progressively as a function of RSI, as visualised in Fig. 13. For descriptive statistics of Experiment 4, please see Table 14 in the supplementary material. A full overview of the MANOVA results is provided in Table 15 in the supplementary material.

#### Error rates

Mirroring the results for response times, the main effect of RSI shows that more errors were made following short compared to long RSIs, *F*(3, 71) = 24.94, *p* < *.*001, η_p_^2^ = .51. S-R binding and retrieval, as indicated by the interaction of stimulus and response, were observed in the error rates, *F*(1, 73) = 22.59, *p* < *.*001, η_p_^2^ = .24,* M*_*PRCs*_ = 5.43% *SD*_*PRCs*_ = 16.07%. There was also a tendency that more errors were made in trials with partial repetitions compared to full or no repetitions in the 500 ms RSI compared to longer RSIs (2,000 ms, 4,000 ms, 5,500 ms). The three-way interaction stimulus, response and RSI missed the significance threshold, *F*(3, 71) = 2.62, *p* = *.*06, η_p_^2^ = .10, however, descriptively it shows a similar pattern as results for response times, as visualised in Fig. 12.

PRCs significantly differed from zero for the 500 ms RSI (*t*(73) = 4.07, *p* < .001, *d*_*z*_ = 0.47), but not for the 2,000 ms RSI(t(73) = 1.89,p= .13,*d*_*z*_= 0.22), the 4,000 ms RSI (t(73) = 2.06,p= .13,*d*_*z*_= 0.24), or the 5,500 ms RSI (t(73) = 2.06,p= .13,*d*_*z*_= 0.24, visualised in Fig. 13). For descriptive statistics of Experiment 4, please see Table 16 in the supplementary material. A full overview of the MANOVA results is provided in Table 17 in the supplementary material.

### Discussion

In Experiment 4, we combined temporal adverbials with an RSI manipulation to enhance the perception of temporal distance (indicated by the adverbials) by associating it with an actual temporal distance. However, even then, there was no segmenting effect of temporal adverbials on PRCs, providing no evidence for a modulation of event-file retrieval. The lack of a moderating effect by temporal adverbial in Experiment 4 adds further evidence to the tentative conclusion that creating an event boundary with a mental representation of time (by means of a temporal adverbial) does not have the same effect on the retrieval of event files as the physical passing of time. However, it should be noted that there was a main effect of temporal adverbial showing longer response times after the adverbial referring to a long period and shorter response times after the adverbial referring to a short period for an otherwise identical RSI (i.e., 3,000 ms) which was not present in Experiment 3. The fact that participants slowed down after encountering temporal adverbials indicating longer periods of time, as compared to those indicating shorter periods, can be interpreted as evidence adverbials were processed. This pattern is consistent with research on temporal orienting, which has shown that symbolic cues directing attention to a moment in time can modulate response preparation (Coull & Nobre, 1998; Nobre, 2001). The memory checks used in Experiment 4 indicate high recall accuracy (mean *M* = 84.3% *SD* = 11.2%) which suggests retrieval of the meaning of the adverbials.

Even though the specific temporal adverbials have been successfully employed in paradigms on event segmentation (Ezzyat & Davachi, 2011), the current studies used a simpler and more abstract narrative setting with a rigid sequence of information presentation and timings as well as repetitions during the experimental phase. Although the stimuli were embedded within a framing story, it could be argued that the repetitive sentences we used lacked sufficient narrative richness and coherence for participants to fully engage with the narrative context.

## General discussion

In this series of experiments, we investigated the effect of temporal factors on the retrieval of event files. Derived from theoretical similarities between the EST (Zacks, 2020) and BRAC framework (Frings et al., 2020), it was assumed that the after-effects of binding should be smaller after temporal factors indicating a long period compared to temporal factors indicating a short period of time. Additionally, we aimed at examining whether event files can be segmented by a semantic reference to the duration of a time interval (Experiment 3 and 4) via temporal adverbials, as previously reported in episodic memory studies on the role of event segmentation (Ezzyat & Davachi, 2011). Across all experiments, the data of the interval effects are more consistent with a decay-based account than with an event segmentation account.

Throughout the experimental series, we found significant after-effects of event file binding and retrieval. However, we did not find any effects of temporal factors on the retrieval of event files when “duration” was instantiated symbolically, that is, by temporal adverbials. In fact, the Bayes factors showed anecdotal evidence in Experiment 3 and moderate evidence in favour of the null hypothesis in Experiment 4, providing no support for a segmenting role of temporal adverbials. Moreover, PRCs, and by inference the likelihood of S-R retrieval, were still not affected when adverbials were additionally paired with corresponding on average shorter or longer time intervals to foster the association that the long adverbial takes in fact longer than the short adverbial and vice versa (Experiment 4). In contrast, results for temporal manipulations in the form of time intervals (Experiment 1, as well as the exploratory analyses in Experiment 4) consistently showed that time intervals of 5,000 ms in Experiment 1 and of at least 2,000 ms in Experiment 4 reduced PRCs, indicating reduced retrieval of event files. The pattern across experiments suggests that only veridical time reduces PRCs, whereas symbolic references to time do not.

There are two possible explanations for the lack of adverbial effects on S-R binding and retrieval processes in Experiments 3 and 4. First, event segmentation (as described in EST) and event-file processing may represent distinct mechanisms and are therefore not necessarily influenced by the same factors, in this case, the linguistic reference to time. However, previous empirical evidence has demonstrated effects of segmentation cues, via context manipulations, on feature binding and retrieval processes (Moeller et al., 2025; Qiu et al., 2023), suggesting at least some overlap between the two frameworks. An alternative explanation might be that not all segmentation factors exert the same degree of influence. This has been shown within the EST framework: goal and time shifts were more potent segmentation cues than spatial or causal shifts (Pettijohn & Radvansky, 2016). Experiment 2 demonstrated that the adverbials used in the present study can effectively segment events when using a segmentation task very similar to those used in event segmentation studies (for example, Zacks et al., 2009). It is possible that the temporal adverbial initiated a memory flush in Experiment 3 and 4. In other words, the cue may have been subjectively perceived as an event boundary, yet the memory of the preceding episode remained accessible. Alternatively, the absence of an effect might stem from the time required to unpack the cue’s semantic meaning, which may exceed the temporal window in which binding effects typically occur. Nevertheless, feature binding has been shown to be influenced by context changes (Moeller et al., 2025; Qiu et al., 2023). These changes consisted of stronger perceptual changes that did not require interpretation of its meaning. Such context effects generally arose within even narrower time windows.

Another consideration concerns the depth to which participants have processed the temporal adverbials in Experiments 3 and 4. Although attention check trial performance confirmed that participants read the adverbials, attention check trials cannot fully ensure that participants engaged deeply with their semantic meaning. In Experiments 3 and 4, the same sentences were used throughout the experiment in a predictable sequence, with little task-relevant information. It could be argued that participants processed the adverbials in a shallow manner, not necessarily constructing or updating an event model based on the given temporal content. However, the temporal adverbials did enter in two-way interactions with both the stimulus and the response factor in Experiment 3 and a main effect of temporal adverbial in Experiment 4 suggesting that the semantic meaning of the adverbial factor levels has been processed. Moreover, memory checks which were used in Experiment 4 revealed performance clearly above chance. This suggests semantic processing of the adverbials but PRCs were still unaffected by temporal adverbials.

With respect to the time-course of after-effects of event files, event files have been reported to be completely decayed after SOAs of 5,000 ms (Hommel & Frings, 2020) but PRCs were still found at 4,000 ms previously (Hommel & Colzato, 2004). We also found no after-effects of event files in Experiment 1 with 5,000 ms RSIs. In our studies, the effect of PRCs in response times already decreased between 500 ms and 1,500 ms and was no longer significant after 2,000 ms in Experiment 4 (exploratory analyses), highlighting that the weakening of trace strength may begin immediately after event file formation and proceed gradually rather than abruptly. Error rates showed the same pattern, with PRCs significant only at the 500 ms RSI, although the corresponding three-way interaction did not reach significance in error rates.

The findings of Experiment 2 are particularly informative in this respect: temporal adverbials did induce event boundaries when segmentation was made task-relevant, yet the same adverbials failed to modulate PRCs in Experiments 3 and 4. This dissociation suggests that event segmentation is not the main mechanism driving the reduction in event file retrieval. That said, the modulating effect of RSI on partial repetition costs in Experiment 1 is well in line with the assumption that the passage of time leads to drifts in (internal) context, thereby increasing the likelihood of perceiving an event boundary the longer the time interval lasts. Taken together, our findings can in principle be accommodated by two accounts: an event segmentation account and a decay-based account. However, it is worth noting that the two accounts are not necessarily mutually exclusive: it might be assumed that information about an ongoing event is maintained in memory to track the achievement of a goal, and that once an event boundary is experienced, active maintenance ceases, and the representation is left to decay. In this view, decay of trace strength might be the consequence of the flushing of the event model described in EST, rather than an independent mechanism. Of the two, the more parsimonious account remains decay: event boundaries appear to be insufficient to reduce trace strength, whereas veridical time weakens the trace gradually and independently of whether a boundary was perceived. Notably, at this point, the BRAC model includes neither decay nor event segmentation as a component (Frings et al., 2020). This decay-based view is consistent with episodic timing theories, which propose that each trial leaves a memory trace representing the stimulus and response events that occurred, and that the strength of this trace decreases over time according to general laws of forgetting, such that more recently formed traces contribute more strongly to subsequent performance than older ones (Los et al., 2014; Salet et al., 2022). In sum, a decay-based account (Hommel & Frings, 2020) better explains the results, whereby the passage of actual time passively reduces the strength of the memory trace representing the event file, making it less accessible for retrieval regardless of whether an event boundary has been experienced. From this perspective, symbolic references to time do not modulate PRCs because they do not alter the actual strength of the underlying memory trace.

With respect to the potential limitations of the study, a key difference to the typical EST research is our study’s adaption of a narrative-based paradigm (Ezzyat & Davachi, 2011) to the S1R1–S2R2 task structure. To create a similarly coherent narrative setting, we replaced the typical abstract stimuli (e.g., uppercase letters, shapes, or sounds) with sentences embedded in a magical tournament storyline. This allowed us to integrate temporal adverbials naturally into the task. Ezzyat and Davachi (2011) probed episodic memory via explicit associative memory tests within coherent narrative settings. The S1R1–S2R2 paradigm, by contrast, does not explicitly require participants to memorize any stimulus- or response-related information during a trial. By embedding the S1R1-S2R2 task within a coherent storyline and incorporating temporal adverbials, we sought to test whether temporal adverbials can influence event segmentation in an implicit way. Although this modification inevitably altered cognitive demands, it provides insight into how event segmentation operates when explicit associative memory is not the primary task.

Another methodological consideration concerns the task’s temporal rigidity. In contrast to most self-paced EST paradigms in which participants pressed a key whenever they experienced a boundary, our design imposed externally controlled timing, ensuring experimental precision but limiting participants’ ability to mentally organise events according to their own temporal flow. While our attention check trials and memory checks confirmed that adverbials were processed, participants may not have had sufficient time or resources to integrate their temporal meaning into an event model. This experimental limitation, however, also highlights an important methodological restriction in EST research: temporal segmentation effects may depend on the degree of autonomy participants have in constructing their own event timeline. Identifying this dependency provides a useful direction for refining other paradigms that manipulate factors such as timing and autonomy.

While these methodological differences limit direct comparability to classical EST paradigms, they also help to understand how temporal and structural factors interact to shape event cognition. Our findings point to the limits under which temporal adverbials modulate event segmentation and offer a basis for future research integrating broader perspectives on event cognition.

## Conclusion

The present research showed that temporal manipulations reduced PRCs, interpreted as reflecting reduced likelihood of event file retrieval, but only when the interval was implemented by means of actual elapsed time. In contrast, references to the symbolic representation of time by temporal adverbials did not affect PRCs, providing no evidence that they created an event boundary affecting event file retrieval. Temporal adverbials also did not segment event files even if they were matched with time intervals of corresponding length to enhance the perception of the temporal distance suggested in these semantic references. Taken together, the pattern of results across Experiments 1, 3 and 4 is more consistent with a decay-based account, in which the passage of veridical time gradually weakens the strength of the memory trace representing an event file, than with an event segmentation account. The finding that temporal adverbials induced event boundaries in Experiment 2 but failed to modulate PRCs in Experiments 3 and 4 is particularly informative in this respect, as it suggests that event segmentation per se is not the mechanism responsible for the reduction in PRCs (interpreted as reflecting reduced event-file retrieval) observed with veridical time intervals. While the cues are in principle capable of generating event boundaries under appropriate conditions, they appear to have no measurable effect at the event file level. Thus, a more parsimonious explanation for the effects of veridical time is decay and needs to be considered in the BRAC framework. These findings raise new questions about whether and under which conditions symbolic temporal information might influence the relation between event segmentation theory and event file processing, which future research will need to address.

## Supplementary information

Below is the link to the electronic supplementary material.

Supplementary file1 (DOCX 58 KB)

## Data Availability

After acceptance of the manuscript, the data and programming code for data analysis of all experiments will be available on [https://www.psycharchives.org]. During the reviewing process, the data and programming code for data analysis are available upon request. The data and the code to reproduce the analyses are available at:Data: https://doi.org/10.23668/psycharchives.22635 Code: https://doi.org/10.23668/psycharchives.22636.

## Footnotes

1 Please note that all post hoc power analyses in this article calculated the statistical power using the a priori assumed effect size rather than the observed effect size.

2 Although data for Experiment 2 were collected after Experiments 3 and 4, it is presented earlier here to facilitate reading flow.

3 ﻿Shortly before publication, we discovered that the data of four participants had been inadvertently omitted from the analyses because the files had been stored in a separate folder. Re-running all analyses with these data files included (applying the same exclusion criteria) did not change the pattern of results or any of our conclusions.

4 This is a deviation from the pre-registration which suggested an exclusion criterion of 50%. However, since that represents chance level only in a binary forced choice task and affected three participants, we decided to increase the criterion to 40% in line with the criteria for R1 and R2.
