# Supplementary material for: Organisation of event files–can temporal factors influence stimulus–response binding and retrieval?
Source: Psychol Res. 2026 Nov 11;90(6):205. doi: 10.1007/s00426-026-02394-3 (PMC13755032; doi:10.1007/s00426-026-02394-3)
Supplement: Supplementary file 1 [file 426_2026_2394_MOESM1_ESM.docx]

| **Table 1** | | | |
| --- | --- | --- | --- |
| *Descriptive statistics (means, standard deviations) for probe response times in milliseconds for Experiment 1 as a function of response, stimulus, and RSI, as well as PRCs in ms by RSI.* | | | |
| **Stimulus** | **Response** | | **PRCs** |
|  | **Repetition** | **Change** |  |
| **500 ms-RSI** | | | |
| Stimulus Change | 806 (163) | 747 (139) | 94 (112) |
| Stimulus Repetition | 742 (155) | 776 (151) |  |
| **1,500 ms-RSI** | | | |
| Stimulus Change | 746 (144) | 721 (121) | 21 (100) |
| Stimulus Repetition | 726 (154) | 722 (146) |  |
| **5,000 ms-RSI** | | | |
| Stimulus Change | 715 (127) | 710 (135) | -13 (111) |
| Stimulus Repetition | 718 (152) | 700 (133) |  |
| *Note.* Standard deviations of the means are shown in parentheses. RSI = Response stimulus interval. PRCs = Partial repetition costs. | | | |

**Table 2**

*3 × 2 × 2 repeated measures MANOVA results for Experiment 1 response times with factors RSI (500 ms, 1,500 ms vs. 5,000 ms), stimulus (S1-S2, repetition vs. change) and response (R1-R2, repetition vs. change).*

| Factor | *df_Num_* | *df_Den_* | *F* | *p* | η^2^_p_ |
| --- | --- | --- | --- | --- | --- |
| (Intercept) | 1 | 63 | 1982.75 | .000 | .97 |
| Stimulus | 1 | 63 | 5.01 | .029 | .07 |
| Response | 1 | 63 | 9.29 | .003 | .13 |
| RSI | 2 | 62 | 34.19 | .000 | .52 |
| Stimulus * Response | 1 | 63 | 19.64 | .000 | .24 |
| Stimulus * RSI | 2 | 62 | 1.23 | .301 | .04 |
| Response * RSI | 2 | 62 | 0.05 | .952 | .00 |
| Stimulus * Response * RSI | 2 | 62 | 19.03 | .000 | .38 |

*Note.* *df_Num_* indicates degrees of freedom numerator. *df_Den_* indicates degrees of freedom denominator. η^2^_p_ indicates partialized eta-squared.

| **Table 3** | | | |
| --- | --- | --- | --- |
| *Descriptive statistics (means, standard deviations) for probe error rates in percent for Experiment 1 as a function of response, stimulus, and RSI, as well as PRCs in ms by RSI.* | | | |
| **Stimulus** | **Response** | | **PRCs** |
|  | **Repetition** | **Change** |  |
| **500 ms-RSI** | | | |
| Stimulus Change | 13 (14) | 4 (6) | 12 (14) |
| Stimulus Repetition | 8 (11) | 10 (9) |  |
| **1,500 ms-RSI** | | | |
| Stimulus Change | 11 (12) | 4 (6) | 7 (14) |
| Stimulus Repetition | 8 (10) | 8 (7) |  |
| **5,000 ms-RSI** | | | |
| Stimulus Change | 8 (12) | 3 (6) | 1 (12) |
| Stimulus Repetition | 6 (9) | 3 (5) |  |
| *Note.* Standard deviations of the means are shown in parentheses. RSI = Response stimulus interval. PRCs = Partial repetition costs. | | | |

**Table 4**

*3 × 2 × 2 repeated measures MANOVA results for Experiment 1 error rates with factors RSI (500 ms, 1,500 ms vs. 5,000 ms), stimulus (S1-S2, repetition vs. change) and response (R1-R2, repetition vs. change).*

| Factor | *df_Num_* | *df_Den_* | *F* | *p* | η^2^_p_ |
| --- | --- | --- | --- | --- | --- |
| (Intercept) | 1 | 63 | 92.46 | .000 | .59 |
| Stimulus | 1 | 63 | 0.06 | .814 | .00 |
| Response | 1 | 63 | 14.34 | .000 | .19 |
| RSI | 2 | 62 | 16.93 | .000 | .35 |
| Stimulus * Response | 1 | 63 | 48.36 | .000 | .43 |
| Stimulus * RSI | 2 | 62 | 1.67 | .197 | .05 |
| Response * RSI | 2 | 62 | 0.08 | .926 | .00 |
| Stimulus * Response * RSI | 2 | 62 | 9.22 | .000 | .23 |

*Note.* *df_Num_* indicates degrees of freedom numerator. *df_Den_* indicates degrees of freedom denominator. η^2^_p_ indicates partialized eta-squared.

**Table 5**

*Descriptive statistics (mean with standard deviations in parentheses) for mean selection probability of perceived event boundary for Experiment 2 as a function of temporal adverbial.*

| **Temporal Adverbial** | | | |
| --- | --- | --- | --- |
|  | **Short** | | **Long** |
| Event Boundary | Probability |  | Probability |
| Cue - S1 | .104 (.267) |  | .055 (.184) |
| S1 - R1 | .033 (.118) |  | .023 (.088) |
| R1 - Adverbial | .296 (.395) |  | .419 (.434) |
| Adverbial - S2 | .188 (.329) |  | .280 (.390) |
| S2 - R2 | .053 (.141) |  | .051 (.126) |
| No Boundary | .326 (.403) |  | .173 (.326) |

*Note.* Cue = Cue for the first response, S1 = first stimulus, S2 = second stimulus, R1 = first response, R2 = second response.

| **Table 6** | | | | |
| --- | --- | --- | --- | --- |
| *Descriptive statistics (means, standard deviations) for probe response times in milliseconds for Experiment 3 as a function of response, stimulus, and temporal adverbial, as well as PRCs in ms by temporal adverbial.* | | | | |
| **Stimulus** | **Response** | | **PRCs** |  |
|  | **Repetition** | **Change** |  |  |
| **Short Adverbial** | | | |  |
| Stimulus Change | 680 (131) | 649 (119) | 21 (89) |  |
| Stimulus Repetition | 675 (136) | 665 (129) |  |  |
| **Long Adverbial** | | | |  |
| Stimulus Change | 681 (127) | 660 (121) | 26 (79) |  |
| Stimulus Repetition | 658 (110) | 663 (134) |  |  |
| *Note.* Standard deviations of the means are shown in parentheses. PRCs = Partial repetition costs. | | | |  |

**Table 7**

*2 × 2 × 2 repeated measures MANOVA results for Experiment 3 response times with factors temporal adverbial (short vs. long), stimulus (S1-S2, repetition vs. change) and response (R1-R2, repetition vs. change).*

| Factor | *df_Num_* | *df_Den_* | *F* | *p* | η^2^_p_ |
| --- | --- | --- | --- | --- | --- |
| (Intercept) | 1 | 69 | 2130.72 | .000 | 0.97 |
| Stimulus | 1 | 69 | 0.47 | .495 | .01 |
| Response | 1 | 69 | 18.70 | .000 | .21 |
| Adverbial | 1 | 69 | 0.41 | .523 | .01 |
| Stimulus * Response | 1 | 69 | 8.91 | .004 | .11 |
| Stimulus * Adverbial | 1 | 69 | 6.70 | .012 | .09 |
| Response * Adverbial | 1 | 69 | 4.30 | .042 | .06 |
| Stimulus * Response * Adverbial | 1 | 69 | 0.18 | .672 | .00 |

*Note.* *df_Num_* indicates degrees of freedom numerator. *df_Den_* indicates degrees of freedom denominator. η^2^_p_ indicates partialized eta-squared.

| **Table 8** | | | | |
| --- | --- | --- | --- | --- |
| *Descriptive statistics (means, standard deviations) for probe error rates in percent for Experiment 3 as a function of response, stimulus, and temporal adverbial, as well as PRCs in ms by temporal adverbial.* | | | | |
| **Stimulus** | **Response** | | **PRCs** |  |
|  | **Repetition** | **Change** |  |  |
| **Short Adverbial** | | | |  |
| Stimulus Change | 8 (9) | 3 (5) | 3 (11) |  |
| Stimulus Repetition | 5 (7) | 5 (6) |  |  |
| **Long Adverbial** | | | |  |
| Stimulus Change | 6 (8) | 3 (5) | 2 (9) |  |
| Stimulus Repetition | 5 (6) | 4 (5) |  |  |
| *Note.* Standard deviations of the means are shown in parentheses. PRCs = Partial repetition costs. | | | |  |

**Table 9**

*2 × 2 × 2 repeated measures MANOVA results for Experiment 3 error rates with factors temporal adverbial (short vs. long), stimulus (S1-S2, repetition vs. change) and response (R1-R2, repetition vs. change).*

| Factor | *df_Num_* | *df_Den_* | *F* | *p* | η^2^_p_ |
| --- | --- | --- | --- | --- | --- |
| (Intercept) | 1 | 69 | 70.59 | .000 | .51 |
| Stimulus | 1 | 69 | 1.40 | .240 | .02 |
| Response | 1 | 69 | 19.40 | .000 | .22 |
| Adverbial | 1 | 69 | 1.94 | .169 | .03 |
| Stimulus * Response | 1 | 69 | 10.28 | .002 | .13 |
| Stimulus * Adverbial | 1 | 69 | 0.00 | .953 | .00 |
| Response * Adverbial | 1 | 69 | 0.24 | .628 | .00 |
| Stimulus * Response * Adverbial | 1 | 69 | 1.08 | .302 | .02 |

*Note.* *df_Num_* indicates degrees of freedom numerator. *df_Den_* indicates degrees of freedom denominator. η^2^_p_ indicates partialized eta-squared.

| **Table 10** | | | | |
| --- | --- | --- | --- | --- |
| *Descriptive statistics (means, standard deviations) for probe response times in milliseconds for Experiment 4 as a function of response, stimulus, and temporal adverbial, as well as PRCs in ms by temporal adverbial.* | | | | |
| **Stimulus** | **Response** | | **PRCs** |  |
|  | **Repetition** | **Change** |  |  |
| **Short Adverbial** | | | |  |
| Stimulus Change | 730 (137) | 694 (120) | 36 (86) |  |
| Stimulus Repetition | 713 (142) | 714 (130) |  |  |
| **Long Adverbial** | | | |  |
| Stimulus Change | 745 (145) | 704 (125) | 38 (89) |  |
| Stimulus Repetition | 727 (150) | 724 (130) |  |  |
| *Note.* Standard deviations of the means are shown in parentheses. PRCs = Partial repetition costs. | | | |  |

**Table 11**

*2 × 2 × 2 repeated measures MANOVA results for Experiment 4 response times with factors temporal adverbial (short vs. long), stimulus (S1-S2, repetition vs. change) and response (R1-R2, repetition vs. change).*

| Factor | *df_Num_* | *df_Den_* | *F* | *p* | η^2^_p_ |
| --- | --- | --- | --- | --- | --- |
| (Intercept) | 1 | 73 | 2369.79 | .000 | .97 |
| Stimulus | 1 | 73 | 0.16 | .689 | .00 |
| Response | 1 | 73 | 18.58 | .000 | .20 |
| Adverbial | 1 | 73 | 7.85 | .006 | .10 |
| Stimulus * Response | 1 | 73 | 27.06 | .000 | .27 |
| Stimulus * Adverbial | 1 | 73 | 0.00 | .097 | .00 |
| Response * Adverbial | 1 | 73 | 0.30 | .585 | .00 |
| Stimulus * Response * Adverbial | 1 | 73 | 0.01 | .923 | .00 |

*Note.* *df_Num_* indicates degrees of freedom numerator. *df_Den_* indicates degrees of freedom denominator. η^2^_p_ indicates partialized eta-squared.

|  | **Table 12** | | | | |
| --- | --- | --- | --- | --- | --- |
|  | *Descriptive statistics (means, standard deviations) for probe error rates in percent for Experiment 4 as a function of response, stimulus, and temporal adverbial, as well as PRCs in ms by temporal adverbial.* | | | | |
| **Stimulus** | | **Response** | | **PRCs** |  |
|  | | **Repetition** | **Change** |  |  |
| **Short Adverbial** | | | | |  |
| Stimulus Change | | 8 (11) | 4 (6) | 4 (13) |  |
| Stimulus Repetition | | 6 (6) | 6 (7) |  |  |
| **Long Adverbial** | | | | |  |
| Stimulus Change | | 10 (9) | 5 (6) | 3 (10) |  |
| Stimulus Repetition | | 6 (7) | 4 (5) |  |  |
|  | | | | |  |

**Table 13**

*2 × 2 × 2 repeated measures MANOVA results for Experiment 4 error rates with factors temporal adverbial (short vs. long), stimulus (S1-S2, repetition vs. change) and response (R1-R2, repetition vs. change).*

| Factor | *df_Num_* | *df_Den_* | *F* | *p* | η^2^_p_ |
| --- | --- | --- | --- | --- | --- |
| (Intercept) | 1 | 73 | 138.54 | .000 | .65 |
| Stimulus | 1 | 73 | 10.33 | .002 | .12 |
| Response | 1 | 73 | 21.40 | .000 | .23 |
| Adverbial | 1 | 73 | 0.12 | .727 | .00 |
| Stimulus * Response | 1 | 73 | 12.87 | .001 | .15 |
| Stimulus * Adverbial | 1 | 73 | 6.15 | .015 | .08 |
| Response * Adverbial | 1 | 73 | 2.32 | .132 | .03 |
| Stimulus * Response * Adverbial | 1 | 73 | 0.29 | .591 | .00 |

*Note.* *df_Num_* indicates degrees of freedom numerator. *df_Den_* indicates degrees of freedom denominator. η^2^_p_ indicates partialized eta-squared.

| **Table 14** | | | | |
| --- | --- | --- | --- | --- |
| *Descriptive statistics (means, standard deviations) for probe response times in milliseconds for Experiment 4 as a function of response, stimulus, and RSI, as well as PRCs in ms by RSI.* | | | | |
| **Stimulus** | **Response** | | **PRCs** |  |
|  | **Repetition** | **Change** |  |  |
| **500 ms-RSI** | | | |  |
| Stimulus Change | 836 (170) | 803 (151) | 102 (187) |  |
| Stimulus Repetition | 791 (170) | 859 (173) |  |  |
| **2,000 ms-RSI** | | | |  |
| Stimulus Change | 751 (157) | 712 (127) | 33 (124) |  |
| Stimulus Repetition | 726 (152) | 721 (128) |  |  |
| **4,000 ms-RSI** | | | |  |
| Stimulus Change | 721 (134) | 701 (126) | 11 (141) |  |
| Stimulus Repetition | 720 (139) | 711 (137) |  |  |
| **5,500 ms-RSI** | | | |  |
| Stimulus Change | 722 (126) | 710 (128) | 2 (141) |  |
| Stimulus Repetition | 714 (133) | 704 (125) |  |  |
| *Note.* Standard deviations of the means are shown in parentheses. RSI = Response stimulus interval. PRCs = Partial repetition costs. | | | |  |

**Table 15**

*4 × 2 × 2 repeated measures MANOVA results for Experiment 4 response times with factors RSI (500 ms, 2,000 ms, 4,000 ms vs. 5,500 ms), stimulus (S1-S2, repetition vs. change) and response (R1-R2, repetition vs. change).*

| Factor | *df_Num_* | *df_Den_* | *F* | *p* | η^2^_p_ |
| --- | --- | --- | --- | --- | --- |
| (Intercept) | 1 | 73 | 2907.70 | .000 | .98 |
| Stimulus | 1 | 73 | 0.11 | .746 | .00 |
| Response | 1 | 73 | 2.03 | .158 | .03 |
| RSI | 3 | 71 | 34.92 | .000 | .60 |
| Stimulus * Response | 1 | 73 | 16.66 | .000 | .19 |
| Stimulus * RSI | 3 | 71 | 0.55 | .651 | .02 |
| Response * RSI | 3 | 71 | 5.00 | .003 | .17 |
| Stimulus * Response * RSI | 3 | 71 | 4.83 | .004 | .17 |

*Note.* *df_Num_* indicates degrees of freedom numerator. *df_Den_* indicates degrees of freedom denominator. η^2^_p_ indicates partialized eta-squared.

| **Table 16** | | | | |
| --- | --- | --- | --- | --- |
| *Descriptive statistics (means, standard deviations) for probe error rates in percent for Experiment 4 as a function of response, stimulus, and RSI, as well as PRCs in ms by RSI.* | | | | |
| **Stimulus** | **Response** | | **PRCs** |  |
|  | **Repetition** | **Change** |  |  |
| **500 ms-RSI** | | | |  |
| Stimulus Change | 16 (16) | 8 (11) | 12 (25) |  |
| Stimulus Repetition | 11 (13) | 15 (16) |  |  |
| **2,000 ms-RSI** | | | |  |
| Stimulus Change | 7 (11) | 5 (8) | 4 (18) |  |
| Stimulus Repetition | 5 (9) | 7 (8) |  |  |
| **4,000 ms-RSI** | | | |  |
| Stimulus Change | 6 (11) | 4 (10) | 3 (13) |  |
| Stimulus Repetition | 4 (8) | 5 (10) |  |  |
| **5,500 ms-RSI** | | | |  |
| Stimulus Change | 5 (7) | 3 (6) | 3 (13) |  |
| Stimulus Repetition | 3 (7) | 3 (7) |  |  |
| *Note.* RSI = Response stimulus interval. PRCs = Partial repetition costs. | | | |  |

**Table 17**

*4 × 2 × 2 repeated measures MANOVA results for Experiment 4 error rates with factors RSI (500 ms, 2,000 ms, 4,000 ms vs. 5,500 ms), stimulus (S1-S2, repetition vs. change) and response (R1-R2, repetition vs. change).*

| Factor | *df_Num_* | *df_Den_* | *F* | *p* | η^2^_p_ |
| --- | --- | --- | --- | --- | --- |
| (Intercept) | 1 | 73 | 170.34 | .000 | .70 |
| Stimulus | 1 | 73 | 0.00 | .990 | .00 |
| Response | 1 | 73 | 1.96 | .166 | .03 |
| RSI | 3 | 71 | 24.94 | .000 | .51 |
| Stimulus * Response | 1 | 73 | 22.59 | .000 | .24 |
| Stimulus * RSI | 3 | 71 | 1.29 | .286 | .05 |
| Response * RSI | 3 | 71 | 0.78 | .507 | .03 |
| Stimulus * Response * RSI | 3 | 71 | 2.62 | .057 | .10 |

*Note.* *df_Num_* indicates degrees of freedom numerator. *df_Den_* indicates degrees of freedom denominator. η^2^_p_ indicates partialized eta-squared.
